# Synthesis and potential applications of cyclodextrin-based metal–organic frameworks: a review

**DOI:** 10.1007/s10311-022-01509-7

**Published:** 2022-09-19

**Authors:** Yang Xu, Ahmed K. Rashwan, Ahmed I. Osman, Eman M. Abd El-Monaem, Ahmed M. Elgarahy, Abdelazeem S. Eltaweil, Mirna Omar, Yuting Li, Abul-Hamd E. Mehanni, Wei Chen, David W. Rooney

**Affiliations:** 1 Department of Food Science and Nutrition, Zhejiang-Egypt Joint Laboratory for Comprehensive Utilization of Agricultural Biological Resources and Development of Functional Foods, Zhejiang University, Hangzhou, 310058 China; 2 Department of Food and Dairy Sciences, Faculty of Agriculture, South Valley University, Qena, 83523 Egypt; 3 School of Chemistry and Chemical Engineering, Queen’s University Belfast, Belfast, BT9 5AG Northern Ireland UK; 4 Chemistry Department, Faculty of Science, Alexandria University, Alexandria, Egypt; 5 Environmental Chemistry Division, Environmental Science Department, Faculty of Science, Port Said University, Port Said, Egypt; 6 Ningbo Research Institute, Zhejiang University, Ningbo, 315100 China; 7 The Cancer Hospital of the University of Chinese Academy of Sciences (Zhejiang Cancer Hospital), Institute of Basic Medicine and Cancer (IBMC), Chinese Academy of Sciences, Hangzhou, Zhejiang China; 8 Department of Food Science and Nutrition, Faculty of Agriculture, Sohag University, Sohag, 82524 Egypt

**Keywords:** Cyclodextrin, Metal–organic frameworks, Synthesis methods, Cyclodextrin-based metal–organic framework applications

## Abstract

Metal–organic frameworks are porous polymeric materials formed by linking metal ions with organic bridging ligands. Metal–organic frameworks are used as sensors, catalysts for organic transformations, biomass conversion, photovoltaics, electrochemical applications, gas storage and separation, and photocatalysis. Nonetheless, many actual metal–organic frameworks present limitations such as toxicity of preparation reagents and components, which make frameworks unusable for food and pharmaceutical applications. Here, we review the structure, synthesis and properties of cyclodextrin-based metal–organic frameworks that could be used in bioapplications. Synthetic methods include vapor diffusion, microwave-assisted, hydro/solvothermal, and ultrasound techniques. The vapor diffusion method can produce cyclodextrin-based metal–organic framework crystals with particle sizes ranging from 200 nm to 400 μm. Applications comprise food packaging, drug delivery, sensors, adsorbents, gas separation, and membranes. Cyclodextrin-based metal–organic frameworks showed loading efficacy of the bioactive compounds ranging from 3.29 to 97.80%.

## Introduction

**Fig. 1:**
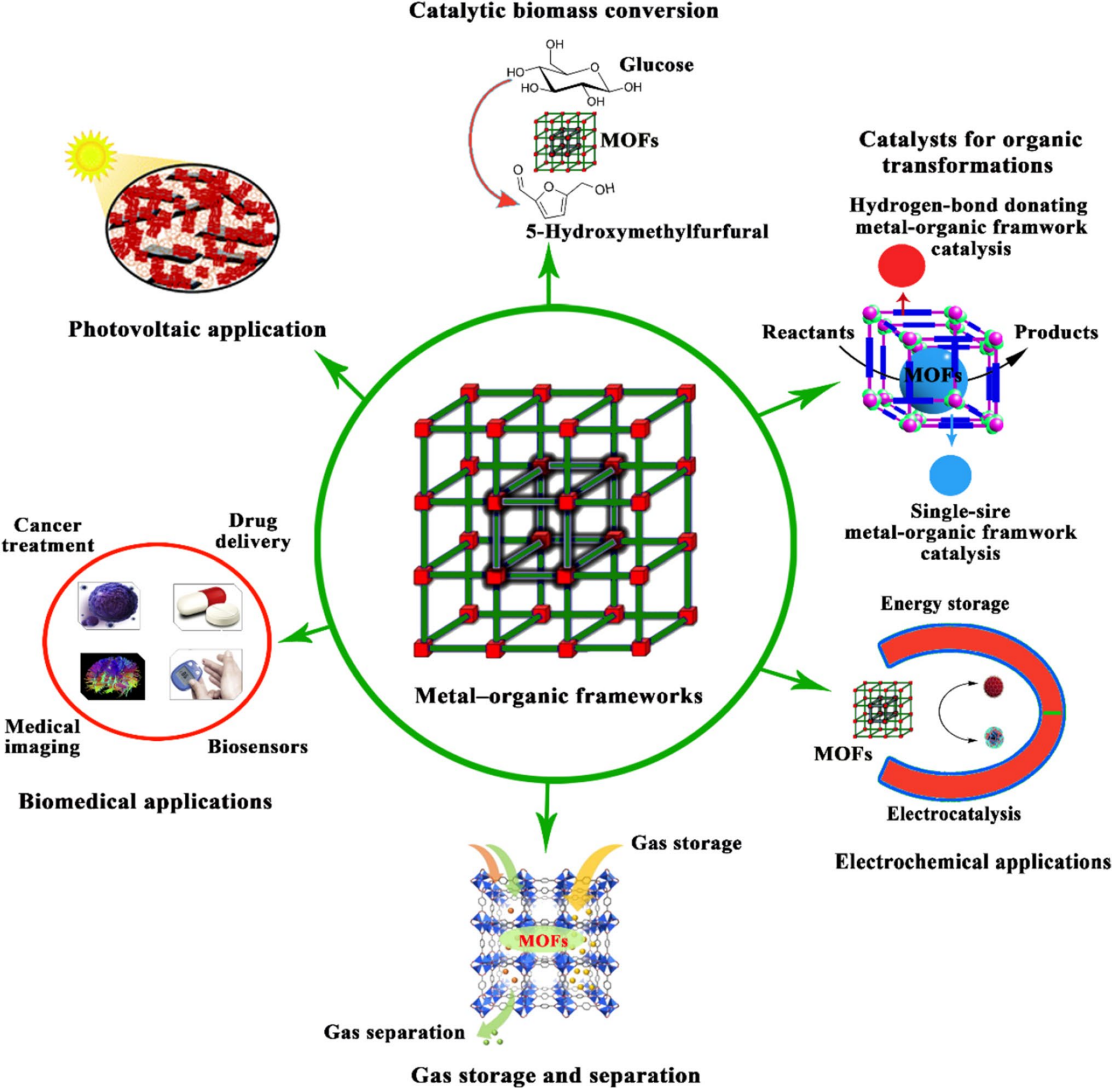
Potential applications of metal–organic frameworks. Metal–organic frameworks can be used in photovoltaic applications. Metal–organic frameworks can be used for electrochemical applications through energy storage and electrocatalysis. Metal–organic frameworks can be used to store and separate the gas. Metal–organic frameworks can be used in biomedical applications such as drug delivery, cancer treatment, and medical imaging. Metal–organic frameworks can be used as catalysis for organic transformations. MOFs refers to metal–organic frameworks. Kreno et al. 2012; Guo et al. 2021; Kaur et al. 2016; Li et al. 2018; Dhakshinamoorthy et al. 2018

Metal–organic frameworks are porous polymeric materials formed by linking metal ions with organic bridging ligands in the most fundamental sense. Metal–organic frameworks have the advantages and properties of both organic and inorganic porous materials and are considered a new development in the interface of molecular coordination chemistry and materials science (Wang et al. 2019; Smaldone et al. 2010; López et al. 2021). Several synthesis techniques such as hydro/solvothermal, mechanochemistry, microwaves, electrochemistry, and sonochemistry can be used to synthesize metal–organic framework (Stolar and Užarević 2020; Wang et al. 2020). Due to the extraordinary physical and chemical properties of metal–organic frameworks, such as their high surface areas, tunable pore sizes, and elastic internal surface properties, metal–organic frameworks have attracted a great deal of researchers’ attention in various applications as presented in Fig. 1 (Zhou and Kitagawa 2014; He et al. 2019a; Sultana et al. 2022). Fields among these applications are sensors (Kreno et al. 2012; Wang et al. 2021a), catalysts for organic transformations (Guo et al. 2021), biomass conversion (Guo et al. 2021; Osman et al. 2021a), photovoltaic (Kaur et al. 2016), electrochemical applications (Xiao et al. 2020), gas storage and separation (Li et al. 2018), photocatalysis (Dhakshinamoorthy et al. 2018), and biomedical applications (Yang and Yang 2020).

**Fig. 2:**
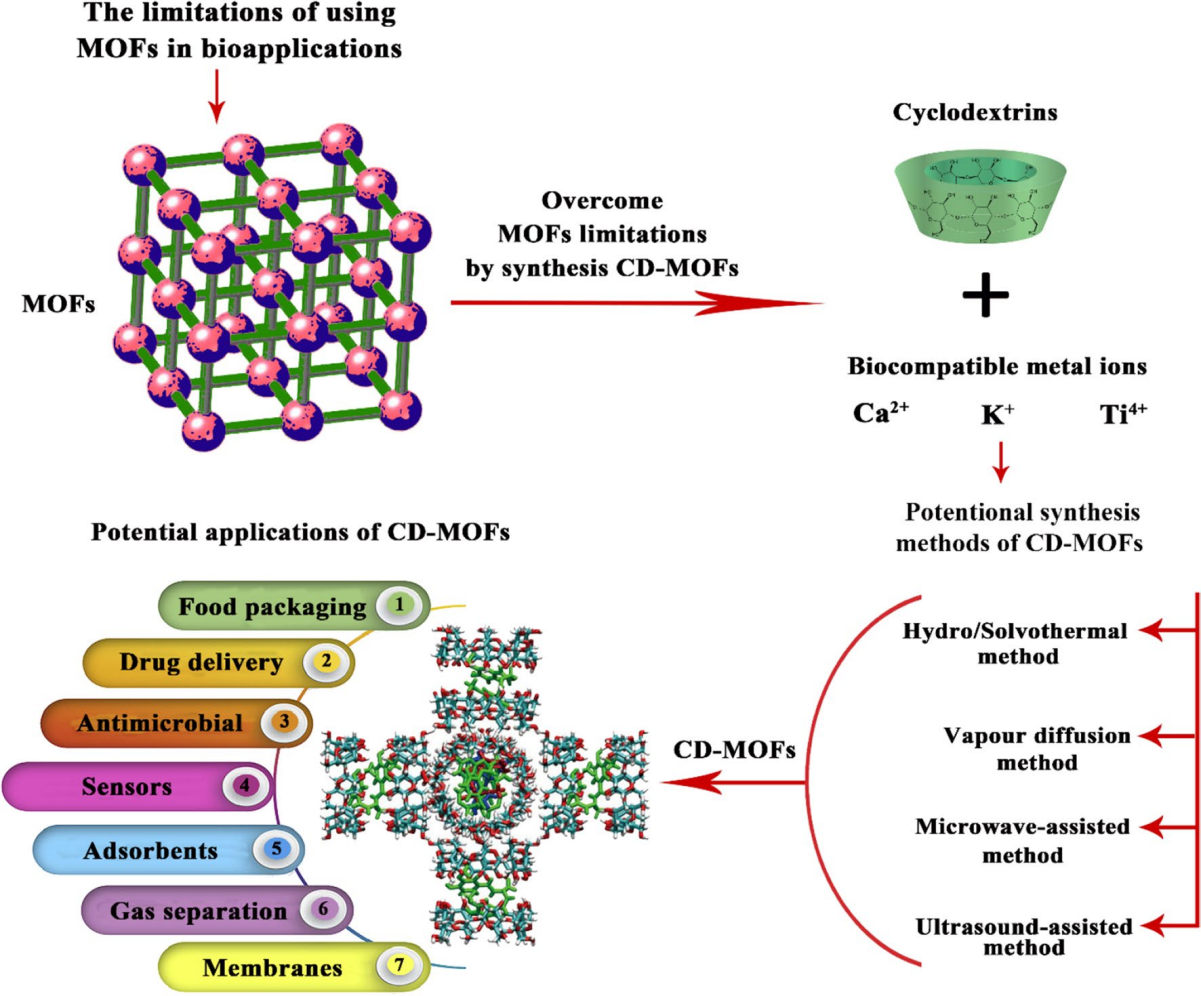
The use of metal–organic frameworks in bioapplications faces some limitations, e.g., the high toxicity of synthetic components, the high toxicity of chemical reagents, and the unrecyclable preparation materials of metal–organic frameworks. In the most fundamental sense, metal–organic frameworks are porous polymeric materials formed by linking metal ions with organic bridging ligands. By constructing metal–organic frameworks from cyclodextrin and biocompatible metal ions, the limitations of metal–organic frameworks in bioapplications can be overcome. Methods for producing cyclodextrin-based metal–organic frameworks include vapor diffusion, microwave-assisted, hydro/solvothermal, and ultrasound-assisted. Food, anticritical, drug delivery, sensors, adsorbents, gas separation, and membranes are some of the applications for cyclodextrin-based metal–organic frameworks. MOFs refers to metal–organic frameworks, and CD-MOFs refers to cyclodextrin-based metal–organic frameworks

However, there are some unfavorable factors, including the high toxicity of synthetic components, e.g., metal ions and organic linkers, or the high toxicity of selected chemical reagents, as well as none of the preparation processes for metal–organic frameworks are recyclable, which pose a substantial obstacle to using metal–organic frameworks in bioapplications, e.g., food and pharmaceutical applications (Fig. 2) (Yang and Yang 2020; Guan et al. 2021; Rajkumar et al. 2019). Therefore, the synthesis of metal–organic frameworks using a biologically acceptable range of biocompatible metal ions such as calcium, potassium, and titanium with peptides, carbohydrates, amino acids, and cyclodextrin derivatives as organic ligand linkers can promote the green production of metal–organic frameworks (Smaldone et al. 2010; Agafonov et al. 2022; Chen et al. 2022; Chen et al. 2021a). Cyclodextrins have greatly interested in producing cyclodextrin-based metal–organic frameworks due to their biocompatibility and structural strength. Cyclodextrins provide metal–organic frameworks with both mechanical strength and flexibility without altering the metal–organic framework’s intrinsic properties. Moreover, cyclodextrins tend to form complexes with metal ions in metal–organic frameworks and interfere with the crystallization process to increase metal–organic framework crystallites (Salgaonkar et al. 2019; Liu et al. 2022; Osman et al. 2021b).

Therefore, this review aims to assess critically and comprehensively the structure and properties of cyclodextrin and metal–organic frameworks. Besides, the recent advances in synthesizing cyclodextrin-based metal–organic frameworks. Moreover, evaluating the potential applications of cyclodextrin-based metal–organic frameworks in several fields, including food, drug delivery, sensors, adsorbents, gas separation, and membrane applications.

## Cyclodextrins

Cyclodextrins are a class of cyclic oligosaccharides produced from starch biodegradation using α-glucanotransferase enzyme, which have been used for decades to increase the solubility and bioavailability of insoluble drugs. Cyclodextrins have several advantages: water-soluble, tasteless, colorless, odorless, non-caloric, non-toxic, biodegradable, biocompatible, and non-carcinogenic (Rajkumar et al. 2019; Chen et al. 2022; Cid-Samamed et al. 2022; Suvarna et al. 2022).

**Fig. 3:**
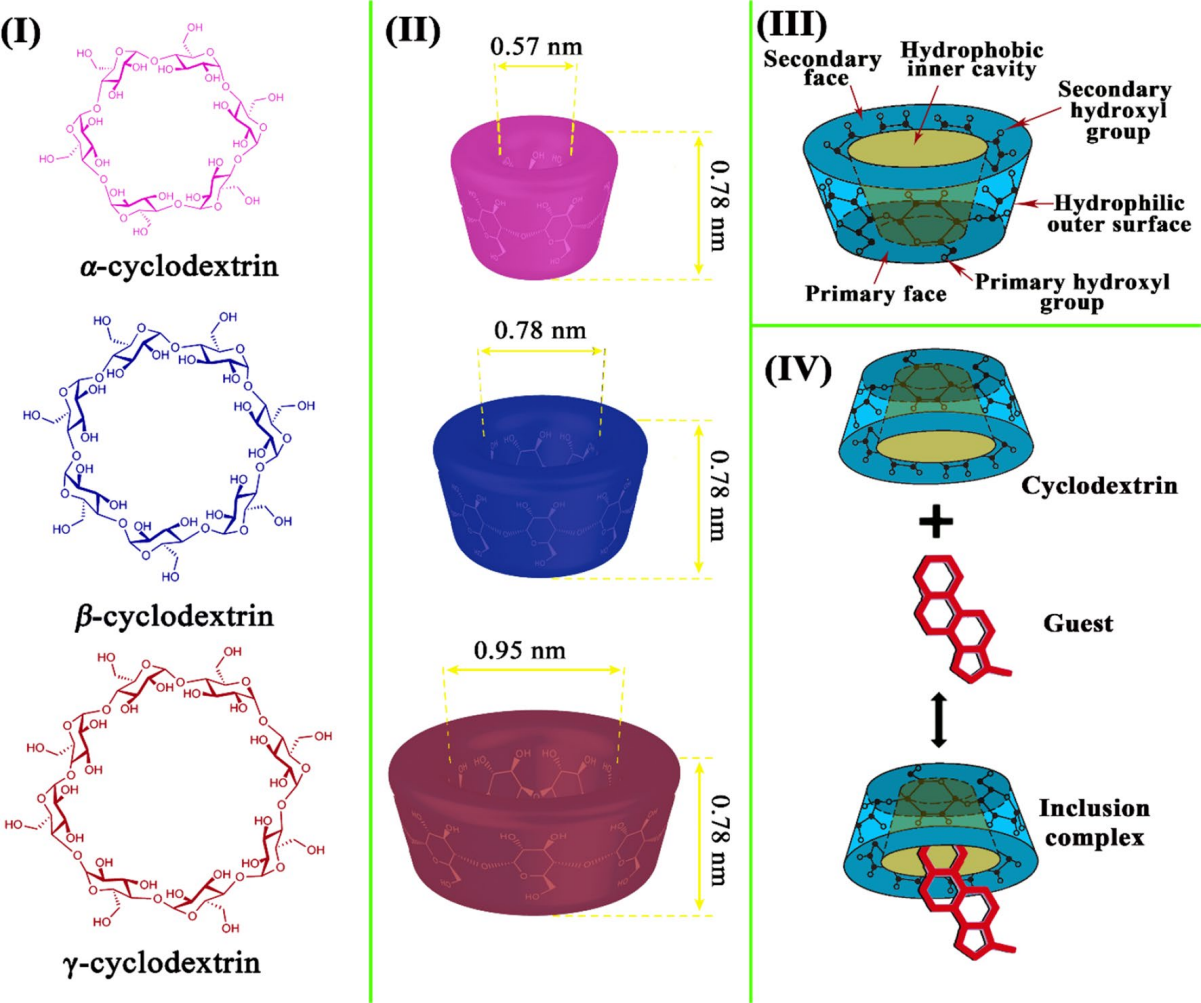
(I) General structure of α-cyclodextrin, β-cyclodextrin, and γ-cyclodextrin, (II) Tridimensional structure of α-cyclodextrin, β-cyclodextrin, and γ-cyclodextrin with different sizes, (III) Digital structure of cyclodextrins, (IV) Digital representation of inclusion complex formation (Rajkumar et al. 2019; Crini 2014). Cyclodextrins have three types, including α-cyclodextrin, β-cyclodextrin, and γ-cyclodextrin. Cyclodextrin contains two faces, e.g., primary face and secondary face, two hydroxyl groups, a hydrophilic outer surface, and a hydrophobic cavity. Cyclodextrin has different sizes of hydrophobic inner cavities based on cyclodextrin type, including 0.57 nm for α-cyclodextrin, 0.78 nm for β-cyclodextrin, and 0.95 nm for γ-cyclodextrin. γ-cyclodextrin has a big hydrophobic inner cavity; thus, γ-cyclodextrin can encapsulate a high amount of bioactive agents. Cyclodextrins can be used as host–guest delivery systems

**Fig. 4:**
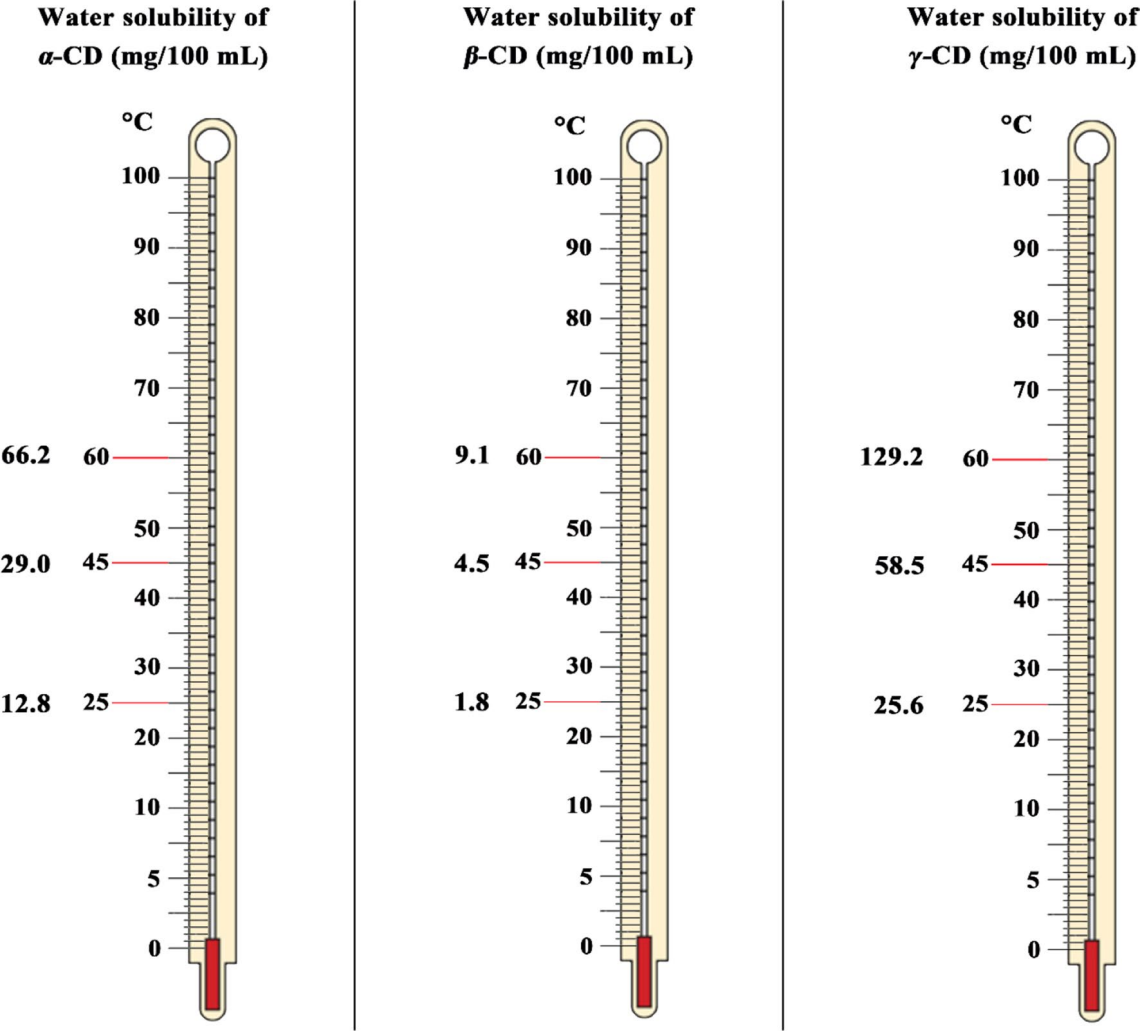
Solubility of α-cyclodextrin, β-cyclodextrin, and γ-cyclodextrin in water under different temperatures including 25, 45, and 60 °C (Poulson et al. 2022). Cyclodextrins can be dissolved in water. α-cyclodextrin can be dissolved in water by 12.8, 29.0, and 66.2 mg/100 mL at 25, 45, and 60 °C, respectively. β-cyclodextrin can be dissolved in water by 1.8, 4.5, and 9.1 mg/100 mL at 25, 45, and 60 °C, respectively. γ-cyclodextrin can be dissolved in water by 25.6, 58.5, and 129.2 mg/100 mL at 25, 45, and 60 °C, respectively. γ-cyclodextrin has a higher water solubility among the three cyclodextrin types. α-CD refers to α-cyclodextrin, β-CD refers to β-cyclodextrin, and γ-CD refers to γ-cyclodextrin

Cyclodextrins consist of six, seven, or eight D-glucopyranose units linked together by α-1,4 glycosidic bonds in the form of hollow cylindrical stereocyclic structures, called α-cyclodextrin, β-cyclodextrin, and γ-cyclodextrin, respectively, with a hydrophilic outer surface and hydrophobic inner cavity (Fig. 3) (Agafonov et al. 2022; Chen et al. 2021a; Chen et al. 2021b; Petitjean et al. 2021). The hydrophobic inner cavity diameters of α-cyclodextrin, β-cyclodextrin, and γ-cyclodextrin range from 4.7 to 5.3, 6.0 to 6.5, and 7.5 to 8.3 Å, respectively (Wankar et al. 2020). α-cyclodextrin, β-cyclodextrin, γ-cyclodextrin, and their hydrophilic derivatives are virtually non-toxic upon orally administered because of their low bioavailability. Interestingly, cyclodextrins can be dissolved in water (Fig. 4) and are less susceptible to enzymatic degradation due to the specific steric structure of glucose units in their structure. Therefore, cyclodextrins can work as promising encapsulation systems and important complexation agents for a broad range of more lipophilic guest molecules and enhance their dispersibility and chemical stability (Hu et al. 2022a; Nguyen et al. 2022; Jansook et al. 2018).

The inclusion of a guest in a cavity of cyclodextrin as a host occurs via replacing the included water molecules with the less polar guest. In which the operation is vigorously favored by the interplay of the solvated hydrophobic cavity of the cyclodextrin with the guest molecule (Cid-Samamed et al. 2022; Young et al. 2012). The solubility, stability, control volatility, dissolution rate, sublimation, and bioavailability of guest molecules have been improved after the inclusion of guest molecules in a cyclodextrin, such as cyclodextrin inclusion complexes (Hu et al. 2022a; Cui et al. 2012; Fernandes et al. 2018; Saldanha do Carmo et al. 2017). Importantly, the cytotoxicity effects of cyclodextrins are negligible, which are considered key attributes for using the ingredients in food applications, cosmetics, drug carriers, textiles, extraction and separation of bioactive compounds, environment protection, and catalysis (Wankar et al. 2020; Nguyen et al. 2022; Fernandes et al. 2018).

Moreover, the production of cyclodextrins reached over 10,000 metric tons/year, where around 70, 15, 5, and 10% of which are β-cyclodextrin, α-cyclodextrin, γ-cyclodextrin, and other cyclodextrin derivatives, respectively (Hu et al. 2022a; Jansook et al. 2018). Therefore, cyclodextrins have attracted the great attention of many researchers to use them in many industrial products, technologies, producing green metal–organic frameworks, and analytical methods (Chen et al. 2021a; Cid-Samamed et al. 2022; Hu et al. 2022a; Nguyen et al. 2022). For instance, encapsulation of curcumin using succinylated-cyclodextrin not only enhanced the stability of curcumin against long-time storage, pasteurization, and ultraviolet-irradiation but also significantly increased curcumin stability toward body temperature, physiological salt concentrations, stomach, and intestinal pH (Hu et al. 2022a). This result may be because curcumin trapped in the hydrophobic cavity of the succinylated-cyclodextrin was protected from physiological conditions (Hu et al. 2022a).

To summarize, cyclodextrin showed several good features such as water solubility, non-toxic, biodegradable, biocompatible, and non-carcinogenic, which is considered a key for using cyclodextrin in several fields. Besides, cyclodextrin has a hydrophilic outer surface and hydrophobic inner cavity; thus, cyclodextrin can be used to deliver both hydrophilic and hydrophobic compounds. Among cyclodextrin types, γ-cyclodextrin has a big hydrophobic inner cavity; thus, cyclodextrin can encapsulate a high amount of bioactive agents.

## Metal–organic frameworks

**Fig. 5:**
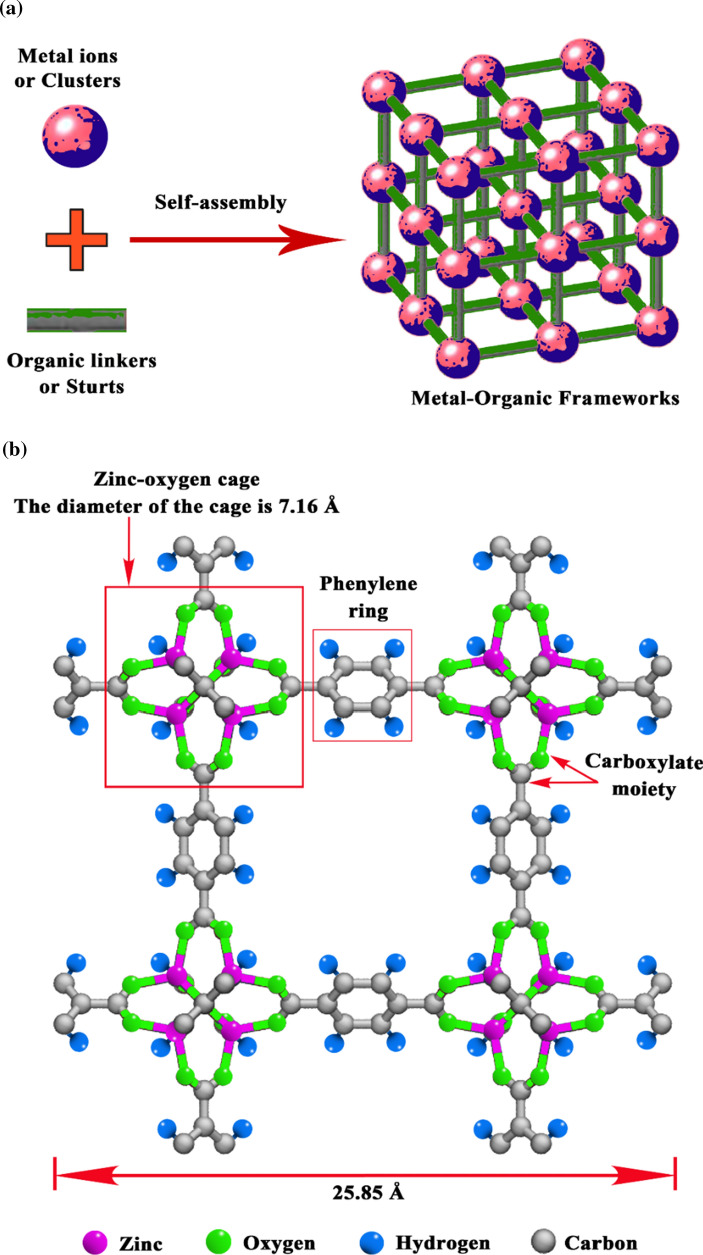
(a) Basic structure of metal–organic frameworks and (b) the cubic structure of metal–organic framework-5. Metal–organic frameworks are produced via self-assembly of metal ions or clusters and organic linkers or struts. Metal–organic framework-5 contains zinc, oxygen, hydrogen, and carbon. Metal–organic framework-5 has a zinc-oxygen cage with a 7.16 Å diameter. Metal–organic framework-5 has a phenylene ring and carboxylate moiety. The diameter of metal–organic framework-5 is 25.85 Å

Metal–organic frameworks, also known as porous coordination polymers, have surfaced as a novel category of versatile materials due to their tunable structure, high specific surface areas, and controllable functionality. Metal–organic frameworks are formed by the connection of metal- or clusters-containing nodes with rigid multipodal organic linkers through strong bonds, using both coordination chemistry and materials science (Fig. 5) (Wang et al. 2019; He et al. 2019a; Xiao et al. 2020; Sharanyakanth and Radhakrishnan 2020).

The structures and features resulting from cyclodextrin-based metal–organic frameworks mainly depend on their synthesis methods and other parameters involved, such as temperature, solvent, reaction time, pH, and pressure (Sharanyakanth and Radhakrishnan 2020). Based on the information from metal–organic frameworks studies, metal–organic frameworks can be constructed using several techniques, including the sonochemical synthesis method, conventional solution method, diffusion synthesis method, ionothermal synthesis method, microwave synthesis method, electrochemical synthesis method, and solvothermal synthesis method (Wang et al. 2019; Wang et al. 2020; Zhou and Kitagawa 2014; Kreno et al. 2012; Guo et al. 2021; Yang and Yang 2020; Sharanyakanth and Radhakrishnan 2020).

Because of metal–organic frameworks’ unique and intriguing structural properties, their synthesis has attracted considerable scientific attention during the last two decades in different scientific fields, as presented in Fig. 1 (Sultana et al. 2022; Kreno et al. 2012; Kaur et al. 2016). For example, capsaicin hydrophobicity was significantly regulated after being loaded in Fe^III^-hollow metal–organic framework-5, and the phase separation problem was effectively solved by Fe^III^-hollow metal–organic framework-5. Besides, adding capsaicin-loaded Fe^III^-hollow metal–organic framework-5 into gelatin/chitosan film significantly improved the tensile strength, water vapor permeability, and ultraviolet barrier properties. Moreover, capsaicin-Fe^III^-hollow metal–organic framework-5 endowed efficient antimicrobial activity to gelatin/chitosan films against *Escherichia coli* compared to gelatin/chitosan films with free-capsaicin (Zhao et al. 2020a). The facilitation of water solubility of the gelatin/chitosan films after adding capsaicin-Fe^III^-hollow metal–organic framework-5 may be because of the strong water absorption by Fe^III^-hollow metal–organic framework-5. In which the water molecules tended to enter inside the polymer matrix and destroy the interaction force between polymer molecules (Zhao et al. 2020a).

Furthermore, the possible explanation for improving water vapor permeability of gelatin/chitosan films after incorporating capsaicin-Fe^III^-hollow metal–organic framework-5 is because of the hydrophilic property of Fe^III^-hollow metal–organic framework-5, which is preferred to attract water molecules despite the presence of capsaicin. Where the porous Fe^III^-hollow metal–organic framework-5 nanocage functioned as a water vapor channel, and more amount of capsaicin-Fe^III^-hollow metal–organic framework-5 made up more water vapor channels favorable for water vapor transport (Zhao et al. 2020a). Yang et al. (2018) studied the adsorption and/or removal of illegal food dyes using a zirconium-based metal–organic framework from a contaminated aqueous solution. Yang et al. (2018) found that the maximum adsorption capacities of Malachite green and Congo red from contaminated aqueous solution using a zirconium-based metal–organic framework reached 357.3 and 1236.9 mg/g, respectively, which are higher than that of the most reported adsorbents (Yang et al. 2018).

However, the real bio-application of metal–organic frameworks still have limitation because many studies reported the toxicity of metal–organic frameworks. For example, metal–organic framework-199 inhibited seedling growth of *Pisum sativum* L. at high concentrations, where the total photosynthesis capability was reduced. Besides, chlorophyll fluorescence damaged the acceptor side of photosystem II after using metal–organic framework-199. The main reason is that cyclodextrin-based metal–organic frameworks-199 released Cu^2+^ in the nutrient solution, led to Cu^2+^ accumulations in seedlings and promoted oxidative stress. Moreover, the photosynthetic inhibitions of metal–organic framework-199 were stronger than equivalent concentrations of Copper (II) nitrate, indicating that metal–organic framework-199 particles also contributed to the environmental hazards (Guan et al. 2021). Therefore, the synthesis of metal–organic frameworks with/using renewable and biodegradable materials such as polysaccharides/oligosaccharides, especially cyclodextrin, can overcome the limitation of applying metal–organic frameworks in bioapplications (Rajkumar et al. 2019; Chen et al. 2022; Sha et al. 2016).

In conclusion, metal–organic frameworks can be used in applications such as sensors, catalysts for organic transformations, biomass conversion, photovoltaic, electrochemical applications, gas storage and separation, and photocatalysis. However, using metal–organic frameworks in bioapplications faces some limitations, e.g., the high toxicity of synthetic components, chemical reagents, and the unrecyclable preparation materials of metal–organic frameworks. Thus, the design of metal–organic frameworks from cyclodextrin and biocompatible metal ions can overcome the limitations of metal–organic frameworks in bioapplications.

## Cyclodextrin-based metal–organic frameworks

Cyclodextrin-based metal–organic frameworks are composed of metal ions epically biocompatible metal ions, e.g., calcium, potassium, titanium, silver, iron, and yttrium with cyclodextrin, e.g., α-cyclodextrin, β-cyclodextrin, and γ-cyclodextrin through well-organized metal–ligand coordination bonds (Fig. 2) (Smaldone et al. 2010; Sha et al. 2016; Forgan et al. 2012). Among cyclodextrin types, the researchers considered γ-cyclodextrin to be suitable for preparing metal–organic frameworks with biocompatible and non-toxic properties. This is because of the presence of single bond binding groups such as –OCCO– in the primary and secondary faces of γ-cyclodextrin, which can be readily used to form complexes with alkali and alkaline earth metal ions (Rajkumar et al. 2019). These structural advantages of γ-cyclodextrin do not exist or are noticed in other cyclodextrin kinds, including α- and β-cyclodextrins. Moreover, the structural features of the α-cyclodextrin-based metal–organic framework and β-cyclodextrin-based metal–organic framework don’t have regular cubic three-dimensional structures such that those for γ-cyclodextrin-based metal–organic frameworks; thus, their morphologies exhibited relatively non-regular shapes compared to the cubic γ-cyclodextrin-based metal–organic frameworks. γ-cyclodextrin-based metal–organic frameworks are also considered the first porous crystals with amphiphilic nanopores (Chen et al. 2021a; Jia et al. 2019; Zhou et al. 2020a; Qiu et al. 2020a).

Cyclodextrin-based metal–organic frameworks are expected to exhibit excellent controllable aerodynamic performance due to their huge surface area and ordered porous interior. Besides, cyclodextrin-based metal–organic frameworks have a normal cubic shape and uniformly distributed spherical voids of 1.7 nm in diameter with an aperture of 0.78 nm (Zhou et al. 2020a). Interestingly, the particle size of porous cyclodextrin-based metal–organic framework crystals can be controlled within a smaller particle size range of 1–10 μm with highly uniform and regular morphology (Liu et al. 2017a; Hu et al. 2019). Therefore, cyclodextrin-based metal–organic frameworks can be used as versatile, including encapsulation of biological agents and transformation of crystal drugs into the molecular state, which will enhance the solubility and bioavailability of hydrophobicity and poorly soluble drugs (Sha et al. 2016; He et al. 2019b; Xu et al. 2019).

Importantly, the inhibitory effects of 5-fluorouracil exhibited higher cytotoxicity against HepG2 cells, while α-cyclodextrin, K_3_(C_36_H_60_O_30_)_2_·7H_2_O, and (C_36_H_60_O_30_)·H_2_O showed no cytotoxicity under the same drug concentration. In which the inhibitory effective cell 50% lethal concentration of 5-fluorouracil, α-cyclodextrin, K_3_(C_36_H_60_O_30_)_2_·7H_2_O, and (C_36_H_60_O_30_)·H_2_O against HepG2 cells was 0.025, 0.1923, 10.0586 and 12.7548 nmol/mL, respectively, indicating that K_3_(C_36_H_60_O_30_)_2_·7H_2_O and (C_36_H_60_O_30_)·H_2_O are a type of renewable, friendly of environmental and biocompatibility drug carrier material (Sha et al. 2016). The toxicity study exhibited that the exposition of Caco-2 and HepG2 cells to different concentrations of γ-cyclodextrin-based metal–organic frameworks for 24 h had no cytotoxicity up to a concentration of 2000 µg/mL in both mammalian cell lines tested. Moreover, the treatment of cells using different concentrations of γ-cyclodextrin-based metal–organic frameworks showed no negative effect on cell viability rate, indicating that the treatments with γ-cyclodextrin-based metal–organic frameworks did not affect the behavior of the cells (Abuçafy et al. 2018).

Another study by Zhou et al. (2020b) showed that cyclodextrin-based metal–organic frameworks had a negligible cytotoxic effect on A549 and Calu-3 cells. Additionally, cyclodextrin-based metal–organic frameworks did not affect lung function and induced inflammatory effects in the lungs (Zhou et al. 2020b). Thus, cyclodextrin-based metal–organic frameworks have recently attracted considerable attention due to their edible, renewable and biodegradable nature. Many studies try to find suitable techniques for preparing cyclodextrin-based metal–organic frameworks with favorable properties such as negligible cytotoxic effect, water-solubility, bioavailability, edibility, renewability, biodegradability, and so on (Wang et al. 2021a; Jia et al. 2019; Zhou et al. 2020a; Xu et al. 2019). Several synthesis methods can be used to prepare different types of cyclodextrin-based metal–organic frameworks as shown in Table 1 and Fig. 2.

**Table 1:** Description, types, advantages, and limitations of common synthesis methods for cyclodextrin-based metal–organic frameworks

Synthesis method	Description	Type of CD-MOFs	Advantages	Limitations	References
Vapor diffusion	The vapor diffusion method is one of the earliest techniques for the synthesis of cyclodextrin-based metal–organic frameworks The conditions used in this diffusion method are mild, requiring only 2–7 days at room temperature (∼25 °C) and pressure	γ-CD-MOF-1 K_2_(C_48_H_80_O_40_)(OH)_2_ Mixed metal Li/K-γ-CD-MOF γ-CD-MOF-2 Rb_2_(C_48_H_80_O_40_)(OH)_2_ γ-CD-MOF-3 Cs_2_(C_48_H_80_O_40_)(OH)_2_ γ-CD-MOF-4 (C_24_H_40_O_20_)(CsOH) (CH_3_OH) Na-α-CD-MOF ([Na(H_2_O)(C_36_H_60_O_30_)]·H_2_O) α-CD-MOF (K_3_(C_36_H_60_O_30_)_2_·7H_2_O)	The simplicity of the synthesis does not need heating Sensitive cyclodextrin-based metal–organic frameworks under mild reaction conditions are synthesized The quality of crystals obtained in this system can be evaluated directly by X-ray diffraction An important application of this method is to concentrate very low volumes of solutions of proteins Vapor diffusion may be useful for the crystallization of proteins that cannot be obtained at high concentrations and may also find applications for concentrating solutions of other samples available in small quantities	High reaction time The vapor diffusion method is difficult to be used to manufacture CD-MOF for large-scale production and future industrial use	(He et al. 2019a; Forgan et al. 2012; Abuçafy et al. 2018)
Microwave-assisted	Microwave-assisted synthesis works based on aligning dipoles of the material in an external field via the excitation produced by microwave electromagnetic radiations and is usually executed in combination with a known synthesis strategy Microwave-assisted synthesis is a potential route to accelerate the synthetic process because of the rapid and selective heating characteristics of microwave-assisted synthesis	CD-MOF-1 γ-CD-MOF-1 K_2_(C_48_H_80_O_40_)(OH)_2_	Microwave-assisted heating can deliver energy instantaneously to targeted components through the interaction of the alternating electromagnetic field with reactants The microwave-assisted technique can lead to a dramatic reduction in synthetic time of metal–organic frameworks from days to minutes Possibility of controlling crystal size Higher yield in remarkable phase purity and phase selectivity Compact synthetic device usage. Synthetic devices with low energy consumption while producing small amounts of chemical waste	The possibility of changing the reaction conditions by regulating the irradiation power is difficult Diverse instruments cannot deliver the same conditions, ultimately hindering reproducibility; therefore, the time of reaction and temperature are also limitations This method requires high-temperature heating, which may limit the popularization of microwave-assisted synthesis	(Sharanyakanth and Radhakrishnan 2020; Liu et al. 2017a; Zhao et al. 2022a)
Hydro/Solvothermal	Hydro/solvothermal synthesis is one of the most typical and effective synthetic methods for constructing nanomaterials with various morphologies, such as cyclodextrin-based metal–organic frameworks This method is carried out using pressure-resistant sealed vessels such as autoclaves or reactors at high ambient pressure greater than 1 atm and temperature greater than 100 °C in the existence of liquids such as water or organic solvent The method is called either a hydrothermal or a solvothermal technique based on the liquid used in the method The method is known as hydrothermal when water is utilized as a reaction medium The method is solvothermal when the nonaqueous solvents are used as a reaction medium	CD-MOF-1 γ-CD-MOF-1 K2(C48H80O40)(OH)2 β-CD-MOF ((C_42_O_35_H_70_)_2_(NaOH)_4_·H_2_O) β-CD-MOF (CD-MOF-1), Cs(OH)·(C_42_H_70_O_35_) β-CD-MOF (CD-MOF-2), [Cs1.5(C_42_H_66_.5O_35_)]_2_ α-CD-MOF ((C_36_H_60_O_30_)·H_2_O)	These synthetic methods offer the advantages of simple, rapid, inexpensive, environment-friendly, and efficient nonconventional heating with high yields Single crystals are readily obtained Single-crystal X-Ray Diffraction is used for structural characterization	Soluble precursors are required Heat and/or aggressive reagents (acids, organic solvents, bases) are needed for regent dissolution Solvent waste generation is high Potentially hazardous handling of explosive/corrosive metal salt, e.g., nitrate/chloride in the presence of organic liquids Waste mineral acids or salts are generated by reactions, e.g., nitric acid and hydrochloric acid Not applicable to heat-sensitive solvents and reagents	(Sharanyakanth and Radhakrishnan 2020; Han et al. 2018; Ding et al. 2019; Kang et al. 2021)
Ultrasound-assisted method	Ultrasound has become an important tool due to the ultrasound applications in the synthesis and modification of nanosized functional inorganic materials Ultrasonic irradiation was introduced for the synthesis of materials with unusual properties Cavitation bubbles grow closer to the solid surface and collapse at a higher amplitude forcing metal ions to bind with cyclodextrin, further accelerating the construction of the cyclodextrin-based metal–organic frameworks system within a short time	α-CD-MOF/Teflon γ-CD-MOF/Teflon β-CD-MOF/Teflon	Fast, environmentally friendly, energy-efficient, room temperature method, nanocrystalline particles synthesis Ultrasonic waves generate vibrations that can create voids that transfer energy to solid particles immersed in the liquid Cavitation generated by ultrasound eases the binding of cyclodextrin molecules with metal ions; thereby, the preparation of cyclodextrin-based metal–organic frameworks can be carried out under mild processing conditions In ultrasound, green solvents can be used to replace toxic organic solvents Cyclodextrin-based metal–organic frameworks obtained by ultrasonic irradiation are thermally more stable than other methods	Sometimes the synthesis temperature near the reactive mixture area cannot be controlled even through a room temperature synthesis method	(Hajra et al. 2021; Shen et al. 2022)

Synthesis methods of CD-MOFs are briefly described. Types of cyclodextrin-based metal–organic frameworks are briefly summarized. The advantages and limitations of cyclodextrin-based metal–organic frameworks synthesis methods are briefly described. CD-MOFs refer to cyclodextrin-based metal–organic frameworks

### Vapor diffusion method

The vapor diffusion method is the first synthetic pathway for preparing and developing different cyclodextrin-based metal–organic frameworks (Smaldone et al. 2010). The vapor diffusion method is liquid–liquid diffusion, where the solvents form two layers based on their densities, the first layer is the precipitant solvent, and the second layer contains the product. Crystal growth also occurs in this method after gradual diffusion of the precipitant solvent into the separate layer, or the reactants in 2 vials with different sizes are gradually diffused via dipping them with physical barriers (Rajkumar et al. 2019; Sha et al. 2016; Hu et al. 2019; Hajra et al. 2021). The γ-cyclodextrin-based metal–organic framework was formulated using reacting γ-cyclodextrin with potassium hydroxide in a hydrous solution, followed by vapor diffusion of methanol into the solution for 6–7 days (He et al. 2019a; Qiu et al. 2020a).

Preparation of γ-cyclodextrin-based metal–organic framework by this methodology requires only room temperature and pressure; however, the particle size of obtained porous cyclodextrin-based metal–organic framework crystals ranges from 200 to 400 μm (Qiu et al. 2020a). This method was modified to control γ-cyclodextrin-based metal–organic framework crystals growth by adding cetyltrimethylammonium bromide, where the addition of cetyltrimethylammonium bromide to the crystallization medium covered the surface of γ-cyclodextrin-based metal–organic framework crystals, slowing down the growth rate and decreased the final crystal size of γ-cyclodextrin-based metal–organic framework (Furukawa et al. 2012). Additionally, the addition of both methanol and cetyltrimethylammonium bromide at the same time to the crystallization medium led to successfully obtaining γ-cyclodextrin-based metal–organic frameworks with a nanoscale range from 200 to 300 nm. However, synthesizing γ-cyclodextrin-based metal–organic frameworks using this method took several days (Furukawa et al. 2012).

Therefore, Liu et al. (2016) successfully decreased the reaction time of synthesizing γ-cyclodextrin-based metal–organic frameworks from 24 to 6 h by developing an effective solvent, e.g., absolute ethanol, methanol, or acetone, via evaporation methodology for synthesizing γ-cyclodextrin-based metal–organic frameworks through setting reaction temperature at 50 °C. Besides, Liu et al. (2016) well-controlled the crystal size of resulting γ-cyclodextrin-based metal–organic frameworks in the range of 5–10 μm by adding 8 mg/mL of cetyltrimethylammonium bromide and around 600 nm by adding both cetyltrimethylammonium bromide and methanol (Liu et al. 2016). However, based on the consideration of green chemistry, there is a problem with using cetyltrimethylammonium bromide in synthesis; cetyltrimethylammonium bromide was quite toxic in the preparation of cyclodextrin-based metal–organic frameworks (Jiang et al. 2021). Therefore, the non-toxic polyethylene glycol 20,000 can be used to replace the toxic cetyltrimethylammonium bromide material, but the disadvantage is that polyethylene glycol 20,000 requires expensive equipment (Jiang et al. 2021). In another study, a novel α-cyclodextrin-based metal–organic framework with chiral helices, K_3_(C_36_H_60_O_30_)_2_·7H_2_O, was synthesized by the vapor diffusion method, which includes infinitely long left-handed helical chains, interdigitated with six circumambient helical chains (Sha et al. 2016).

Moreover, potassium cations-cyclodextrin-based metal–organic frameworks crystals were synthesized by an optimized methanol vapor diffusion method with 141.875 mg β-cyclodextrin and 5.0 mL potassium hydroxide in an aqueous solution. The K-cyclodextrin-based metal–organic frameworks crystals showed high adsorption that was 150.2 and 199.8 mg drug/g potassium cations-cyclodextrin-based metal–organic frameworks when the reaction time was 48 h at both temperatures of 35 and 45 °C (Kong et al. 2019). Modified vapor diffusion methods also were adopted for synthesizing β-cyclodextrin-based metal–organic framework via dissolving β-cyclodextrin and sodium hydroxide in ethanol/water in a sealed vessel. After 2 weeks of reaction, β-cyclodextrin-based metal–organic frameworks were formed, with each K^+^ ion coordinating with 6 atoms of oxygen from the surrounding four β-cyclodextrins, where 2 adjacent β-cyclodextrins were connected by a K^+^ to form the pores (Qiu et al. 2020a). Additionally, β-cyclodextrin-based metal–organic framework-b was successfully prepared by mixing 12.3 mg of potassium hydroxide with 50 mL of distilled water for 10 min at room temperature and 50 mg of β-cyclodextrin through slow vapor diffusion of methanol into the solution in distilled water. Then crystalline products were collected and washed with methanol and chloroform and pure β-cyclodextrin-based metal–organic framework-b crystalline was obtained with a yield of 60% after drying under vacuum (Kang et al. 2021).

To summarize, the vapor diffusion method is the first synthetic route for the preparation and development of various cyclodextrin-based metal–organic frameworks. Using only room temperature and pressure, cyclodextrin-based metal–organic frameworks can be successfully produced by the vapor diffusion method. However, the particle size of the porous cyclodextrin-based metal–organic framework crystals obtained ranges between 200 and 400 µm. With specific additives such as cetyltrimethylammonium bromide or polyethylene glycol as assistant agents, the crystal size of cyclodextrin-based metal–organic frameworks can be controlled in the range of 5–10 µm.

### Hydro or solvothermal method

Hydro/solvothermal synthesis is one of the most typical and effective synthetic methods for constructing nanomaterials with various morphologies, such as cyclodextrin-based metal–organic frameworks. This method is a process carried out using pressure-resistant sealed vessels such as autoclaves or reactors at high ambient pressure greater than 1 atm and temperature greater than 100 °C in the existence of liquids such as water or organic solvent (Xiao et al. 2020; Sharanyakanth and Radhakrishnan 2020; Sha et al. 2016). The method is called either a hydrothermal or a solvothermal technique based on the using liquid; when the water is utilized as a reaction medium, the method is known as the hydrothermal process. While in case the preparation is carried out in the presence of the nonaqueous solvents, the method is termed a solvothermal process (Qiu et al. 2020a; Icten 2021).

For example, metal–organic nanotubes constructed from β-cyclodextrin or γ-cyclodextrin and Pb^2+^ were synthesized through a solvothermal reaction, and cyclodextrin-based metal–organic framework yield ranged from 70 to 80% (Wei et al. 2012a). Additionally, a new β-cyclodextrin-based metal–organic framework-1 was successfully obtained by solvothermal method from the reaction of β-cyclodextrin, sodium oxalate, 10 mL methanol, and water. The mixture was stirred for 1 h at room temperature, then sealed in an 18 mL Teflon-lined reactor and heated at 160 °C for 3 days (Lu et al. 2015). Furthermore, the γ-cyclodextrin-based metal–organic framework was successfully synthesized by hydro/solvothermal process using a mixture of γ-cyclodextrin (324 mg), potassium hydroxide (8 mg), and deionized water (10 mL), then added 12 mL of methanol to the mixture. The final solution was heated at 50 °C for 20 min, then centrifuged and collected the micron-sized cyclodextrin-based metal–organic framework crystals were washed with 15 mL of ethanol and methanol twice, respectively. The crystals were dried overnight at 50 °C under a vacuum. The surface morphology characteristic of γ-cyclodextrin-based metal–organic framework synthesized by hydro/solvothermal process showed a uniform cubic crystal of γ-cyclodextrin-based metal–organic framework (Han et al. 2018).

Besides, Ding et al. (2019) showed that the preparation of porous potassium cations-γ-cyclodextrin-based metal–organic frameworks using hydrothermal synthesis produced 422 g porous potassium cations-γ-cyclodextrin-based metal–organic frameworks/L of mother solution and hydrothermal synthesis yield increased by 19 times compared with solvothermal synthesis, for which the productivity was only 22.5 g/L (Ding et al. 2019). β-cyclodextrin-based metal–organic framework-a was also produced through a solvothermal process by mixing 5 g of β-cyclodextrin, and 1.234 g of potassium hydroxide, then the mixture was milled for 60 min. The resulting product was washed with methanol and chloroform and then dried under a vacuum to obtain β-cyclodextrin-based metal–organic framework-a (yield 98%) (Kang et al. 2021).

To summarize, the hydro/solvothermal method involves using pressure-resistant sealed vessels, such as autoclaves or reactors, at high ambient pressure greater than 1 atm and temperature higher than 100 °C in the presence of liquids like water or organic solvent. By utilizing a hydro/solvothermal process, cyclodextrin-based metal–organic frameworks were successfully developed. Using a hydro/solvothermal process, the yield of cyclodextrin-based metal–organic frameworks can reach 98%.

### Microwave- and ultrasound-assisted method

The microwave- and ultrasound-assisted synthesis method is widely used to synthesize cyclodextrin-based metal–organic frameworks with special morphologies. Besides, microwave- and ultrasound-assisted synthesis method presents the advantages of simple, rapid, inexpensive, environment-friendly, and efficient nonconventional heating with high yields (Qiu et al. 2020a; Shen et al. 2022). γ-cyclodextrin-based metal–organic framework-1 was synthesized using a microwave method, where 324 mg of γ-cyclodextrin and 112 mg of potassium hydroxide were dissolved in 10 mL water with the pre-addition of 6 mL methanol. A clear solution was obtained from the above contents after heating at 10–100 °C via microwave illumination with power: 100 W and time 1–120 min. Then, 256 mg of polyethylene glycol 20,000 was rapidly added to induce crystallization. After isolation and washing crystals for two times with 15 mL ethanol and methanol, the micron-sized γ-cyclodextrin-based metal–organic framework-1 crystals were collected after 1 h. Then, the obtained sample was dried in a vacuum oven at 50 °C overnight (Liu et al. 2017a).

However, the microwave-assisted method conditions require high-temperature heating, which may limit the popularization of the microwave-assisted method. Thus, using the ultrasound-assisted method to prepare cyclodextrin-based metal–organic frameworks could synthesize cyclodextrin-based metal–organic framework crystals with uniform size and morphology via optimizing the ultrasound power, reaction time, and reaction temperature during the synthesis process (Shen et al. 2022; Samuel et al. 2018). The authors successfully prepared a γ-cyclodextrin-based metal–organic framework by ultrasound-assisted rapid synthesis using follows materials; 648 mg of γ-cyclodextrin and 256 mg of potassium hydroxide in 20 mL of ultra-pure water. The clear and transparent solution was ultrasonically processed using a probe of an ultrasonic generator at a frequency of 20 kHz and a power of 540 W, reaction under intermittent action for 10 min, the intermittent ultrasonic action mode is on for 2 s, off for 2 s. After the start of ultrasonication of the solution, 256 mg of polyethylene glycol-8000 was quickly added to trigger the deposition of cyclodextrin-based metal–organic framework crystals. The morphology of the sample obtained under the ultrasonic power of 540 W had a typical uniform cubic state, and the size was about 8 μm (Shen et al. 2022).

In conclusion, microwave- and ultrasound-assisted synthesis offers the benefits of simple, rapid, inexpensive, eco-friendly, and effective non-conventional heating with high yields. The studies demonstrated that cyclodextrin-based metal–organic frameworks could be successfully synthesized with the aid of microwaves and ultrasound. The morphology and size of cyclodextrin-based metal–organic frameworks synthesized using microwave- and ultrasound-assisted synthesis were uniformly cubic and approximately 8 µm.

## Potential applications of cyclodextrin-based metal–organic frameworks

Cyclodextrin-based metal–organic frameworks are one of the most widely used categories of polysaccharide functionalized cyclodextrin-based metal–organic frameworks ascribed to their authentic orderly cavities that originated from the presence of cyclodextrin in the composite ~ 17 Å and the existence of many hydroxyl groups that are ordered and organized to form a cage-like construction (Nadar et al. 2019). Cyclodextrin-based metal–organic framework materials have various structures depending on the cyclodextrin used in the process. Among these materials, the γ-cyclodextrin-based metal–organic framework possesses outstanding adsorption capability that is 10 times as higher as activated carbon, ascribed to the synergistic effect of different interactions and molecular recognition. These properties and features make them excellent candidates for various applications, including gas separation, adsorption, membranes, and sensors (Fig. 2) (Wang et al. 2018).

### Food applications

Typically, the cyclodextrin-based metal–organic frameworks are exemplary carriers for bioactive chemicals, e.g., polyphenols, lipids, flavoring agents, along with others, because cyclodextrin-based metal–organic frameworks are readily synthesized from different renewable sources Table 2. The capacity of the cyclodextrin-based metal–organic framework as carriers to accept insoluble bioactive chemicals is their most appealing property (Blight et al. 2020; Wang et al. 2021b; Zhang et al. 2019a). Hu et al. (2021) employed the encapsulation technique to investigate menthol encapsulation in three types α-cyclodextrin-based metal–organic framework, β-cyclodextrin-based metal–organic framework, and γ-cyclodextrin-based metal–organic framework. Hu et al. (2021) reported that the β-cyclodextrin-based metal–organic framework had the utmost encapsulation effectiveness of 22.54%, with a methanol content of 21.76% (w/w), which was substantially for 3–4 times higher than the other as-used solid moieties such as amylose and V-type starch, attributing to the appropriate pore size and surface area of β-cyclodextrin-based metal–organic framework (Hu et al. 2021). Glycyrrhizic acid is a natural triterpene glycoside with several medicinal properties, including anti-allergenic, antibacterial, anti-inflammatory, and anticancer characteristics. However, glycyrrhizic acid applicability is currently restricted due to glycyrrhizic acid’s exceedingly poor water solubility and loading efficiency (Izutani et al. 2014).

**Table 2:** Cyclodextrin-based metal–organic frameworks are efficient carriers for various bioactive compounds in the food industry

MOF composite	Metal ion	Organic ligand	Target bioactive compound	Loading efficacy	References
β-CD-MOF	K^+^	β-CD	Methanol	22.54%	Guo et al. (2021)
β-CD-MOF	K^+^	β-CD	Quercetin	196.4 mg g^−1^	Kong et al. (2019)
β-CD-MOF	K^+^	β-CD	Anise leaf polyphenols	97.80%	Wang et al. (2021b)
β-CD-MOF	K^+^	β-CD	Emodin	142.2 mg/g	Kong et al. (2019)
γ-CD-MOF	K^+^	γ-CD	Glycyrrhizic acid	850 μg/mg	Qiu et al. (2019)
γ-CD-MOF	K^+^	γ-CD	Curcumin	3.29%	Zhou et al. (2020a)
γ-CD-MOF	K^+^	γ-CD	Resveratrol	21.0%	Qiu et al. (2020b)
γ-CD-MOF	K^+^	γ-CD	Folic acid	35.0%	Xu et al. (2019)
γ-CD-MOF	K^+^	γ-CD	Limonene	0.094 Wt.% γ-CD-MOF/limonene	Zhang et al. (2019a)
γ-CD-MOF	K^+^	γ-CD	Ethyl propionate	0.218 Wt.% γ-CD-MOF/ ethyl propionate	Zhang et al. (2019a)
γ-CD-MOF	K^+^	γ-CD	Myrcene	0.105 Wt.% γ-CD-MOF/myrcene	Zhang et al. (2019a)

Metal–organic frameworks composites and their materials are briefly described. Target bioactive compounds and their loading efficacy in the MOFs composites are summarized. CD-MOF refers to a cyclodextrin-based metal–organic framework, and CD refer to cyclodextrin

Qiu et al. (2019) successfully synthesized uniform nanocrystals of the cyclodextrin-based metal–organic framework via an environmentally pathway of seed-mediated crystallization coupled with sonication. The efficacy of loading glycyrrhizic acid onto a cyclodextrin-based metal–organic framework was recorded. Moreover the findings related to the cell toxicity of safe glycyrrhizic acid-loaded cyclodextrin-based metal–organic framework strongly confirmed their potency as nanocarriers in food applications (Qiu et al. 2019). A well-organized cage-like structure of a γ-cyclodextrin-based metal–organic framework was fabricated through the interaction between γ-cyclodextrin and potassium ions. After that, the water-insoluble bioactive folic acid was efficiently incorporated into the γ-cyclodextrin-based metal–organic framework with a molar ratio of 1:2 γ-cyclodextrin-based metal–organic framework: folic acid.

Interestingly, the as-used Ship-in-Bottle approach increased the perceived solubility of folic acid by 1450 folds. Furthermore, the bioavailability of loaded folic acid in a γ-cyclodextrin-based metal–organic framework is enhanced by a ratio of 1.48 compared with the non-loaded folic acid (Xu et al. 2019). Similarly, the cyclodextrin-based metal–organic framework has been shown as an outstanding carrier for different insoluble bioactive chemicals such as curcumin and resveratrol, which originated from the natural environment by improving their solubility (Zhou et al. 2020a; Qiu et al. 2020b).

However, the bioactive compounds are mostly regarded as strong antioxidants; however, bioactive compounds are chemically unstable and quickly metabolized; therefore, ameliorating their characteristics is of interest. Resveratrol, a naturally a triphenolic phytoalexin molecule derived from various plant types, e.g., grapes, mulberries, and pistachio, has favorable pharmacological activities such as antioxidant, anti-inflammatory, antimicrobial, antiaging, cardioprotective, neuroprotective, chemopreventive, and anticancer properties (Frémont 2000; Neves et al. 2012; Sanders et al. 2000). On the other hand, resveratrol has minimal water solubility and is quickly isomerized and deactivated under different operational conditions, e.g., medium pH, the surrounding temperature, and light (Amri et al. 2012). Qiu et al. (2020b) hypothesized a modest one-step approach for forming cyclodextrin-based metal–organic framework/chitosan nanocapsules between the oppositely charged chitosan and cyclodextrin-based metal–organic framework and further inspected its performance as a delivery route for bioactive resveratrol agent. The results revealed that the encapsulation efficacy of resveratrol onto cyclodextrin-based metal–organic framework/chitosan nanocapsules rose noticeably from 66.5 to 91.3%.

Moreover, the antioxidant activity and photostability of loaded resveratrol were remarkably improved (Qiu et al. 2020b). Ke et al. (2019) developed a nanosized edible-cyclodextrin-based metal–organic framework using a simple vapor diffusion process to encapsulate epigallocatechin gallate. Ke et al. (2019) found that the cyclodextrin-based metal–organic framework-epigallocatechin gallate offered a better antioxidant activity than the non-attached epigallocatechin gallate. Furthermore, the produced cyclodextrin-based metal–organic framework-epigallocatechin gallate inhibited cancer cell proliferation in C6 cells, as demonstrated by the cell viability experiment. This disclosed that such harmless and safe nanoscale cyclodextrin-based metal–organic framework-epigallocatechin gallate porous materials have considerable potential for food and biomedical applications (Ke et al. 2019). Isosteviol is a diterpene chemical that is a synthetic derivative of steviol glycoside. Isosteviol has a wide spectrum of pharmacological effects and biological activities, including antifungal, antiviral, anti-neoplastic, enhancing insulin sensitivity, and lowering plasma triglycerides (Abdullah Al-Dhabi et al. 2015; Carrera-Lanestosa et al. 2017; Gu et al. 2018; Huang et al. 2014; Liu et al. 2017b; Malki et al. 2017; Ruiz-Ruiz et al. 2017).

Moreover, isosteviol also lowers vasoconstriction through ion channel modulation and has a cardioprotective effect against coronary reperfusion damage with little toxicity (Adehin et al. 2019). However, the main disadvantage of using isosteviol as medication might be its limited solubility and bioavailability (Yin et al. 2017). As a model of insoluble drugs, isosteviol was studied and attempted to be loaded into a cyclodextrin-based metal–organic framework using the approach of solvent incubation. In the cyclodextrin-based metal–organic framework, two distinct isosteviol-cyclodextrin-based metal–organic framework loading molar ratios of 0.5:1 and 1:1 were assessed. Furthermore, the oral bioavailability of the isosteviol-cyclodextrin-based metal–organic framework (1:1) was compared to that of the isosteviol-cyclodextrin-based metal–organic framework (0.5:1) and pure isosteviol (Chen et al. 2021b). The isosteviol solubility was less than 20.00 ng/mL at pH 1.0 and 4.5 but rose to 20,074.30 ng/mL at pH 6.8 and 129.58 ng/mL in water with a considerable pH dependence. Additionally, the bioavailability of isosteviol-cyclodextrin-based metal–organic framework (1:1) in rats was 8.67-fold greater than isosteviol and 1.32- and 1.27-fold larger than isosteviol-cyclodextrin and isosteviol-cyclodextrin-based metal–organic framework (0.5:1), respectively (Chen et al. 2021b). Systematically, when isosteviol in the cyclodextrin-based metal–organic framework was found at a low loading ratio, the inclusion process was dominant, whilst the nanocluster mechanism was responsible for improving bioavailability at a high loading ratio (Chen et al. 2021b).

Besides, the crystal stability and loading capacity of alkali-based potassium metal-cyclodextrin-based metal–organic framework toward two drugs of emodin (2.3 mg/ml) and quercetin (3.2 mg/ml) were comprehensively assayed. As a result, the potassium metal-cyclodextrin-based metal–organic framework presented adsorption capacities of 150.2 and 199.8 mg/g for quercetin and emodin, respectively, under running operational parameters of pH = 6, residence interaction time = 48 h, and adsorbent concentration = 30 mg. The adsorption kinetics of tested drugs matched the pseudo-second-order model assumption (chemisorption). Most notably, X-Ray diffraction pattern analysis revealed that the drug loading technique did not impair the material's crystallinity (Kong et al. 2019). Similarly, the stabilities and antioxidant features of polyphenols extracted from Chinese star anise leaves and phenol were largely modulated after inoculation into a β-cyclodextrin-based metal–organic framework and γ-cyclodextrin-based metal–organic framework, respectively (Wang et al. 2021b; Li et al. 2020).

Moreover, the cyclodextrin-based metal–organic framework was investigated as an ideal carrier to improve micronutrient stability, e.g., vitamin A palmitate. Vitamin A palmitate is a commonly used vitamin A derivative that cannot be generated by the human body and must be consumed through food or dietary supplements. It is prone to different chemical reactions of oxidation, polymerization, pyrolysis, dehydration, and decarboxylation, attributing to the presence of numerous conjugated double bonds and an ester bond in the skeleton (Bourassa et al. 2013; Hemery et al. 2015; Pignitter et al. 2014). Zhang et al. (2018) studied the improvement in the stability of vitamin A palmitic by employing a cyclodextrin-based metal–organic framework as a carrier. Compared to commercially supplied vitamin A powder, the microencapsulation efficacy of the prepared γ-cyclodextrin-based metal–organic framework-vitamin A palmitic was 9.77%, with a molar ratio of n-metal–organic frameworks: n-VAP = 3.2:1.0. Significantly, an increment in the stability of vitamin A palmitate microencapsulated by cyclodextrin-based metal–organic framework without the addition of any antioxidant was superior to that of the best available reference product on the market, with a 1.6-fold extended half-life, because of the protective mechanism of a cyclodextrin-based metal–organic framework for vitamin-A palmitic, vitamin-A molecules preferentially coiled within the cavities of dual cyclodextrin pairs in a cyclodextrin-based metal–organic framework. In addition, the cyclodextrin-based metal–organic framework is an effective novel carrier for delivering and protecting vitamin A palmitate in food applications (Zhang et al. 2018).

Regarding the mentioned examples, cyclodextrin-based metal–organic framework carriers could enhance the delivery of bioactive chemicals with poor solubility and their bioavailable characteristic. Furthermore, the internal space and unique cavity of the cyclodextrin-based metal–organic framework can increase bioactive molecule instability and give the possibility of prolonged release of the loaded bioactive component. Because of these benefits, the cyclodextrin-based metal–organic framework has the potential to be used as a carrier of food additives containing bioactive chemicals in the food sector (Shen et al. 2021). Metal–organic framework templates have shown considerable promise in combating bacterial infection. However, metal–organic frameworks may have downsides of possible health concerns. Metal ions must be reduced using either high heat, pressure, or radiation throughout the synthesis process. The cyclodextrin-based metal–organic framework is another option for palatable dextrin molecules with high hydrophilicity. Cyclodextrin-based metal–organic frameworks can be employed as a reduced metal salt due to incorporating numerous hydroxyl groups during their synthesis (Wei et al. 2012b). Silver nanoparticles are a potential new version of traditional antibacterial nano-systems for combating bacterial resistance and filling the medication discovery gap. However, managing their size and colloidal stability, which readily cluster or coalesce in solid and aqueous states, presents many obstacles (Humbatova et al. 2017; Le Ouay and Stellacci 2015).

Shakya et al. (2019) used the water-soluble and biocompatible cyclodextrin-based metal–organic framework in the template-guided synthesis of ultrafine silver nanoparticles. The cyclodextrin-based metal–organic framework-based synthetic method combines silver nitrate reduction and silver nanoparticle immobilization in a single pot, yielding dual benefits of decreased particle size and increased stability (Shakya et al. 2019). Meanwhile, the produced silver nanoparticles are easily dispersible in aqueous environments and effectively suppress bacterial growth. The surface modification of cross-linked cyclodextrin-based metal–organic framework particles with Gly-Arg-Gly-Asp-Ser peptides improves the hemostatic action, which works in tandem with the antibacterial impact to improve wound healing. As a result, the technique of synthesis and immobilization of ultrafine silver nanoparticles in the cyclodextrin-based metal–organic framework in conjunction with Gly-Arg-Gly-Asp-Ser peptides modification shows significant potential for the rational design of effective wound healing devices (Shakya et al. 2019). Luo et al. (2020) demonstrated that the co-delivery of superfine nano-silver with solubilized sulfadiazine employing a cyclodextrin-based metal–organic framework as a carrier showed greater antibacterial effectiveness than insoluble silver sulfadiazine in the current investigation. The abundant hydroxyl moieties in the cyclodextrin-based metal–organic framework were used to reduce silver precursors into 4–5 nm silver nanoparticles that were then trapped within nanosized cavities. The incorporation of sulfadiazine molecules in the hydrophobic cavities of cyclodextrin molecular pairs was promoted by microporous cyclodextrin-based metal–organic framework. The hydrophilic cyclodextrin-based metal–organic framework may easily dissolve inside exudates at the wound site, allowing the medication to be released. This method increased the sulfadiazine water solubility by 50 times, resulting in increased sulfadiazine release and antibacterial action. The minimal inhibition concentration of this composite to *E. coli* and *Staphylococcus aureus* was around 4 mg/mL. The cyclodextrin-based metal–organic framework also inhibited nano-silver particle aggregation, stabilizing particle size and improving particle performance (Luo et al. 2020). This research provided insight into the antibacterial applicability of cyclodextrin-based metal–organic framework composites in food safety.

Cyclodextrin-based metal–organic frameworks have gained popularity due to their superior adsorption properties. As a result, cyclodextrin-based metal–organic frameworks may be employed as chemical sensors to detect toxic compounds and as adsorbents to remove harmful substances from food. Metal–organic framework-prepared chemical sensors have been widely used in food safety (Huangfu et al. 2021). However, many metal–organic framework crystals are hard and fragile, restricting their applicability in chemical sensors. As a result of their high water solubility and biocompatibility, cyclodextrin-based metal–organic frameworks have emerged as promising options in the sensing field (Wei et al. 2012a). Tu et al. (2020) have designed a Mxene/carbon nanohorn/β-cyclodextrin-based metal–organic framework through an electrostatic self-assembly strategy and examined Mxene/carbon nanohorn/β-cyclodextrin-based metal–organic framework as an effective electrochemical sensor for carbendazim pesticides. Mxene/carbon nanotubes had a big specific surface area, many accessible active sites, and a high conductivity, which provided additional mass transport channels and improved the carbendazim mass transfer capacity and catalysis. The Mxene/carbon nanohorn/β-cyclodextrin-based metal–organic framework electrode extended a wide linear range from 3.0 nM to 10.0 μM and a low detection limit of 1.0 nM, referring to the synergistic action of MXene/carbon nanotubes and cyclodextrin-based metal–organic framework (Tu et al. 2020).

Furthermore, the produced sensor displayed great selectivity, repeatability, long-term stability, as well as acceptable application in some vegetables, such as tomato samples (Tu et al. 2020). Antibacterial films and packaging materials in food storage and preservation might be a future research trend. Ground Curcuma rhizome contains curcumin, a significant component of turmeric, a yellow stain commonly used as a spice and food colorant (Sharma et al. 2005). Moreover, curcumin may be introduced as an antioxidant, anti-inflammatory, and anti-carcinogenic molecule (Heger et al. 2014; Murakami et al. 2008). Curcumin has been encapsulated satisfactorily in a cyclodextrin-based metal–organic framework without affecting its crystallinity. Curcumin and cyclodextrin-based metal–organic framework engage strongly via an H bonding interaction between the cyclodextrin hydroxyl group and that of phenolic moieties present in the curcumin (Moussa et al. 2016). The resultant compound, produced in alkaline conditions (pH 11.5), exhibits a maximum absorption peak at ~ 520 nm and a maximum emission peak at 600 nm. Most notably the curcumin stability in this complex was increased by more than threefold compared to their counterpart in non-combined curcumin and curcumin-γ-cyclodextrin (Moussa et al. 2016). These findings clarified a possible safe cyclodextrin-based metal–organic framework technology that may be utilized to store and stabilize curcumin for food applications. Chen et al. (2022) proposed a simple program for synthesizing a novel nanoscale γ-cyclodextrin-based metal–organic framework, further delivering the encapsulation of curcumin. The nano γ-cyclodextrin-based metal–organic framework has not only superb monodispersity and crystalline structure but also a high loading capacity. Nano γ-cyclodextrin-based metal–organic framework significantly improves curcumin solubility and top-down uniform dispersion during dissolution. Additionally, the release behavior of the composite is governed by the loaded amount of curcumin (Chen et al. 2022).

To summarize, the most prevalent food applications for cyclodextrin-based metal–organic frameworks are antibacterial films and food packaging materials. As a result of their renewable construction materials, cyclodextrin-based metal–organic frameworks are ideal carriers for bioactive food additives in the food industry. Encapsulating bioactive food compounds in cyclodextrin-based metal–organic frameworks substantially enhanced their solubility, stability, and bioavailability. Curcumin demonstrated the highest affinity for cyclodextrin-based metal–organic frameworks among all bioactive food compounds.

### Drug delivery applications

Interestingly, cyclodextrin-based metal–organic frameworks’ unparalleled merits have given them immense consideration in drug delivery applications. Consequently, excessive developments have been executed to enhance cyclodextrin-based metal–organic framework performance as drug delivery vehicles. In one attempt, Xue et al. (2019) ameliorated the γ-cyclodextrin-based metal–organic framework via crosslinking with a disulfide-containing linker and removing the K^+^ ion for efficient doxorubicin loading with an auspicious safety profile. The as-fabricated γ-cyclodextrin-based metal–organic framework and modified forms of the γ-cyclodextrin-based metal–organic framework were characterized by bountiful tools, including FE-scanning electron microscopy that inferred the cubic structures of the γ-cyclodextrin-based metal–organic framework, ssCL-cyclodextrin-based metal–organic framework, and novel cubic gel particles. Besides, thermogravimetric analysis patterns showed an enhancement in the thermal behavior of both ssCL-cyclodextrin-based metal–organic framework and novel cubic gel particles compared to the pristine γ-cyclodextrin-based metal–organic framework, which is most likely due to the resultant covalent bonds during the crosslinking process. The stability test in the cell culture medium pointed out the inferior stability of the γ-cyclodextrin-based metal–organic framework since γ-cyclodextrin-based metal–organic framework dissolved after 30 s, while the ssCL-cyclodextrin-based metal–organic framework and novel cubic gel particles exhibited high stability even after 24 h incubation. Notably, the efficient doxorubicin adsorption capacity of novel cubic gel particles and cubic gel particles enhanced with the incubation time until they reached the maximum efficient doxorubicin loading by 45 mg efficient doxorubicin/g of carriers after 40 min (Xue et al. 2019).

In another attempt, Singh et al. (2017) examined the mesoporous diphenyl carbonate-crosslinked γ-cyclodextrin-based metal–organic framework sponges' ability to load efficient doxorubicin. Brunauer–Emmett–Teller measurements signalized that the high specific surface area of diphenyl carbonate-crosslinked-γ-cyclodextrin-based metal–organic framework sponges reached 140 m^2^/g and 315 m^2^/g for micro diphenyl carbonate-crosslinked γ-cyclodextrin-based metal–organic framework and nano diphenyl carbonate-crosslinked γ-cyclodextrin-based metal–organic framework, respectively. Moreover, a propitious efficient doxorubicin loading within both micro- and nano-diphenyl carbonate-crosslinked γ-cyclodextrin-based metal–organic frameworks was 60–80 mg/g incremented with the increase in the crosslinking time from 4 to 24 h. In addition, the loss during the efficient doxorubicin loading process in the cross-linked diphenyl carbonate-crosslinked γ-cyclodextrin-based metal–organic framework within 4, 8, 12, and 24 h were 49.27, 17.38, 11.88, and 5.64 wt%, respectively. A slight loss in the diphenyl carbonate-crosslinked γ-cyclodextrin-based metal–organic framework-24 may be assigned to the expected loss throughout the washing and drying (Singh et al. 2017). In another investigation, Xu et al. (2019) used the Ship-in-Bottle approach for folic acid loading within the cage-like structure γ-cyclodextrin-based metal–organic framework. Folic acid was loaded within the γ-cyclodextrin-based metal–organic framework by three ratios between folic acid: γ-cyclodextrin-based metal–organic framework; 1:1, 1:2, and 2:1. Scanning electron microscopy images showed a regular cubic structure of the γ-cyclodextrin-based metal–organic framework, folic acid-γ-cyclodextrin-based metal–organic framework (1:2), and folic acid-γ-cyclodextrin-based metal–organic framework (1:1), while few particles of folic acid-γ-cyclodextrin-based metal–organic framework (2:1) collapsed, which is most likely due to the excessive amount of the folic acid loading.

In addition, X-Ray Diffraction of the folic acid-γ-cyclodextrin-based metal–organic frameworks revealed that there were no distinguishing peaks to folic acid, indicating the incorporation of folic acid into the molecular level and the formation of interactions between γ-cyclodextrin-based metal–organic framework and folic acid. More importantly, the folic acid loading mechanism within the γ-cyclodextrin-based metal–organic framework was investigated using molecular docking. The folic acid molecular dimensions are 1.98 nm × 0.76 nm, meaning that the folic acid molecules could smoothly diffuse through the γ-cyclodextrin-based metal–organic framework cavities since they are loose enough (Xu et al. 2019). This strongly proved that the folic acid-γ-cyclodextrin-based metal–organic framework system looks like the ship in a bottle-like system, agreeing with a previous study by (di Nunzio et al. 2014). Besides, the diffusion of folic acid into the γ-cyclodextrin-based metal–organic framework cavities occurred via two steps; (I) folic acid molecules distributed into the hydrophobic pores of the γ-cyclodextrin-based metal–organic framework via the hydrophobic, e.g., docking energy = − 10.4 kcal/mol. (II) The excess folic acid molecules were attracted to the hydrophilic pores of the γ-cyclodextrin-based metal–organic framework via the columbic interactions between hydroxyl groups of the γ-cyclodextrin-based metal–organic framework and the hydrophilic group of folic acid (docking energy = − 8.1 kcal/mol). Furthermore, when the number of folic acid molecules in the hydrophilic pores was greater than three, a disordered interaction occurred between the γ-cyclodextrin-based metal–organic framework and folic acid. Thus, the change in the morphology of the folic acid-γ-cyclodextrin-based metal–organic framework (2:1) that was shown from X-Ray Diffraction and scanning electron microscopy may be assigned to the disordered distribution of the extra amount of the loaded folic acid (di Nunzio et al. 2014).

**Fig. 6:**
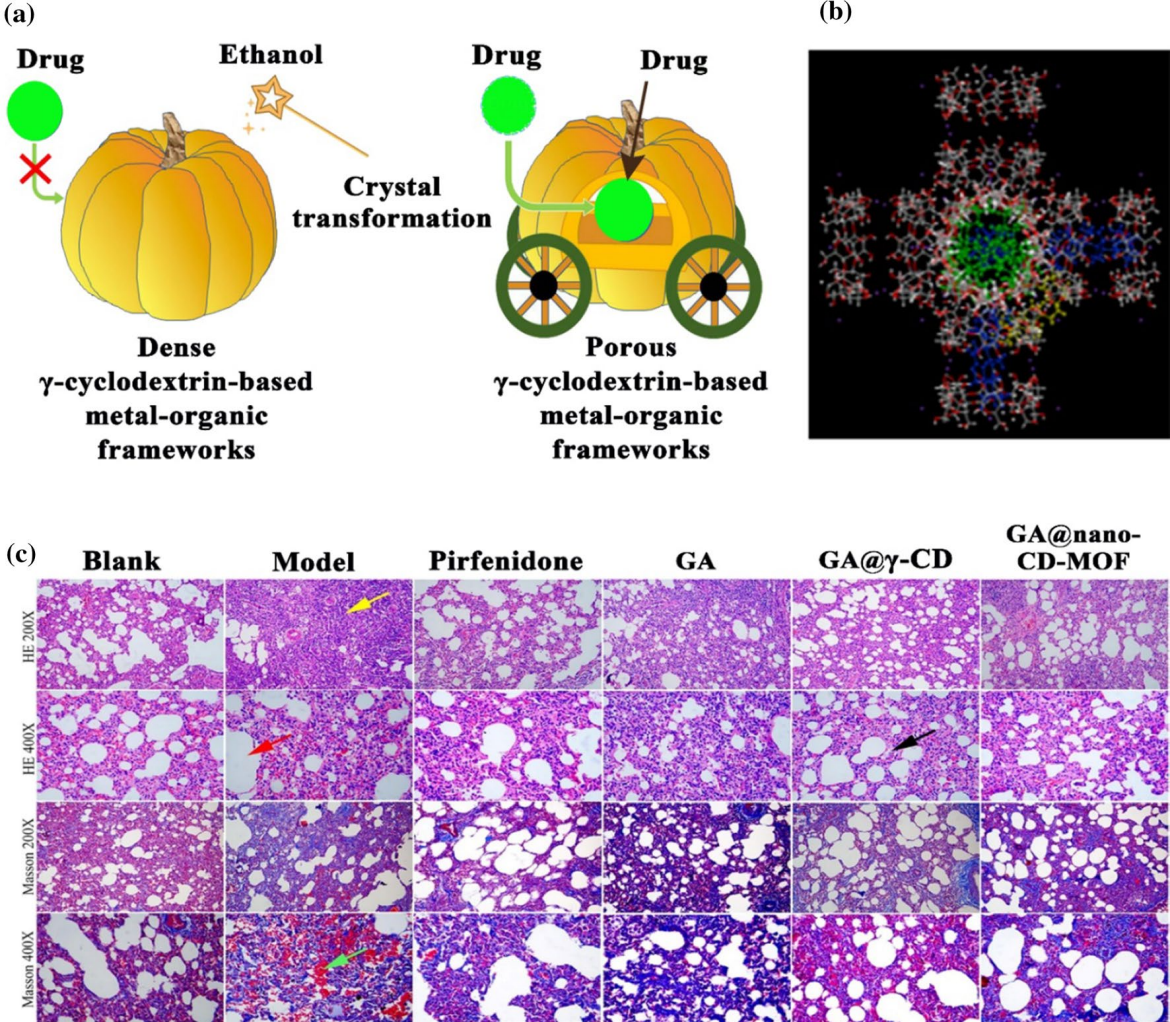
(**A**) Crystal transformation of dense potassium acetate-γ-cyclodextrin-based metal–organic framework to porous potassium acetate-γ-cyclodextrin-based metal–organic framework (Ding et al. 2019), (**B**) The conformations of 18β-glycyrrhetinic acid@nano-γ-cyclodextrin-based metal–organic framework, and (**C**) Graph of pharmacodynamic graph of the treatment of pulmonary fibrosis by 18β-glycyrrhetinic acid (Liu et al. 2022). Copyright, 2022, Elsevier. CD-MOF refers to a cyclodextrin-based metal–organic framework, GA refers to 18β-glycyrrhetinic acid, CD refers to cyclodextrin, GA@nano-CD-MOF refers to 18β-glycyrrhetinic acid@nano-γ-cyclodextrin-based metal–organic framework

In another study, Ding et al. (2019) adopted a novel technique to fabricate a porous γ-cyclodextrin-based metal–organic framework via the crystal transformation of the dense one to enhance the loading capability of valsartan (Fig. 6A). Interestingly, the transformed dense potassium acetate-γ-cyclodextrin-based metal–organic framework had a higher valsartan loading 33.5% than the pure porous potassium acetate-γ-cyclodextrin-based metal–organic framework 30.2%. This result may be explained by the cavities of the porous potassium acetate-γ-cyclodextrin-based metal–organic framework that was filled with air during the drying step, decreasing the carried valsartan into the cavities. Contrariwise, the dense potassium acetate-γ-cyclodextrin-based metal–organic framework arrangement was disintegrated and recombined during the transformation step to form the porous structure, inferring the full contact of valsartan on the dense potassium acetate-γ-cyclodextrin-based metal–organic framework crystals. Hence, the drying process impacted the crystal phase of the dense potassium acetate-γ-cyclodextrin-based metal–organic framework, reflecting the significance of water molecules in shaping the metal–organic framework crystals. The X-Ray Diffraction patterns elucidated the change of the potassium acetate-γ-cyclodextrin-based metal–organic framework crystals from polarized light columnar to amorphous after the freeze-drying step. Notably, the dispersion of the amorphous potassium acetate-γ-cyclodextrin-based metal–organic framework into water transformed again to the dense potassium acetate-γ-cyclodextrin-based metal–organic framework (Ding et al. 2019).

In that respect, Liu et al. (2022) inspected the capability of nano-γ-cyclodextrin-based metal–organic framework to load 18β-glycyrrhetinic acid. The molecular docking between nano-γ-cyclodextrin-based metal–organic framework and glycyrrhetinic acid was scrutinized using AutoDock Vina 1.1.2. Liu et al. (2022) found that the binding free energy values in the cyclodextrin rings, the γ-cyclodextrin-based metal–organic framework cavities, and on the γ-cyclodextrin-based metal–organic framework surface were − 9.3, − 7.8, and − 5.6 kcal/mol, respectively (Fig. 6B) (Liu et al. 2022). These binding free energy verified the strong H-bonding between the o-containing group of glycyrrhetinic acid and the hydroxyl group of the γ-cyclodextrin-based metal–organic framework. Thereby, the glycyrrhetinic acid-loaded molecules were mainly stabilized into the γ-cyclodextrin-based metal–organic framework cavities and the cyclodextrin pairs. This result was consistent with the characterization results of the glycyrrhetinic acid@nano-γ-cyclodextrin-based metal–organic framework. Moreover, the impact of glycyrrhetinic acid treatment was examined on a rat's lung tissues with pulmonary fibrosis (Fig. 6C). The apparent from the blank group was that the normal alveolar morphology and inter-spacing, while the Masson stain elucidated the reticulate alveolar wall without any interstitial hyperplasia. For the glycyrrhetinic acid group, the morphology and the structure of the lung were distributed in addition to the increase in the thickness of the inflammatory cell (Liu et al. 2022). In another attempt, Abuçafy et al. (2018) fabricated various γ-cyclodextrin-based metal–organic frameworks via a vapor diffusion approach for the encapsulation and controlled release of sodium diclofenac.

In general, the vapor diffusion method possesses remarkable advantages, including simplicity, quite high yield, and importantly vapor diffusion method does not consume energy. Besides, the yield of potassium cations-γ-cyclodextrin and sodium-γ-cyclodextrin were 70 and 72%, respectively, agreeing with the study by Smaldone et al. (2010). At the same time, the yield of iron-γ-cyclodextrin was 62% which was smaller than potassium cations-γ-cyclodextrin and sodium-γ-cyclodextrin. The X-Ray Diffraction patterns of potassium cations-γ-cyclodextrin, sodium-γ-cyclodextrin, and iron-γ-cyclodextrin illustrated the relative peaks of the γ-cyclodextrin-based metal–organic framework at 2*θ* = 5.3, 7.4, and 16.7 Å (Forgan et al. 2012; Furukawa et al. 2012). Furthermore, Fourier-transform infrared spectroscopy spectra of the three fabricated γ-cyclodextrin-based metal–organic frameworks signalized the belonging band to the methylene stretching of γ-cyclodextrin between 2850 and 3000 cm^−1^, but narrower than pure γ-cyclodextrin owing to the molecular change after the formation of the metal-γ-cyclodextrin bond. Furthermore, the safety profiles of potassium cations-γ-cyclodextrin, sodium-γ-cyclodextrin, and iron-γ-cyclodextrin were examined by the cytotoxicity test on Caco-2 and HepG2 human cells at a slightly basic pH. The result proved the absence of toxicity of potassium cations-γ-cyclodextrin, sodium-γ-cyclodextrin, and iron-γ-cyclodextrin since the cell viability did not change even when the γ-cyclodextrin-based metal–organic frameworks concentration reached 2000 µg/ml, agreeing with (Tofzikovskaya et al. 2015). The in vitro release of sodium diclofenac after 2 h at pH 1.2 from potassium cations-γ-cyclodextrin, sodium-γ-cyclodextrin, and iron-γ-cyclodextrin were 23, 22, and 20%, respectively, clarifying the low release rate. Then after 5 h, an increase in the drug release was recorded, reaching 70%. While in the basic medium (pH 7.4), the sodium diclofenac release from potassium cations-γ-cyclodextrin, sodium-γ-cyclodextrin, and iron-γ-cyclodextrin after 6 h were 33, 39, and 42%, respectively. Notably, in pH 6.4, the sodium diclofenac release was equal for 8 h, then a noticeable increase was observed since the cumulative % of the sodium diclofenac release during 24 h were 63, 41, and 42% for iron-γ-cyclodextrin, potassium cations-γ-cyclodextrin and sodium-γ-cyclodextrin, respectively. Such behavior of iron-γ-cyclodextrin may be attributed to the higher pores volume that allows to solvent to entrain and release the drug (Tofzikovskaya et al. 2015).

**Fig. 7:**
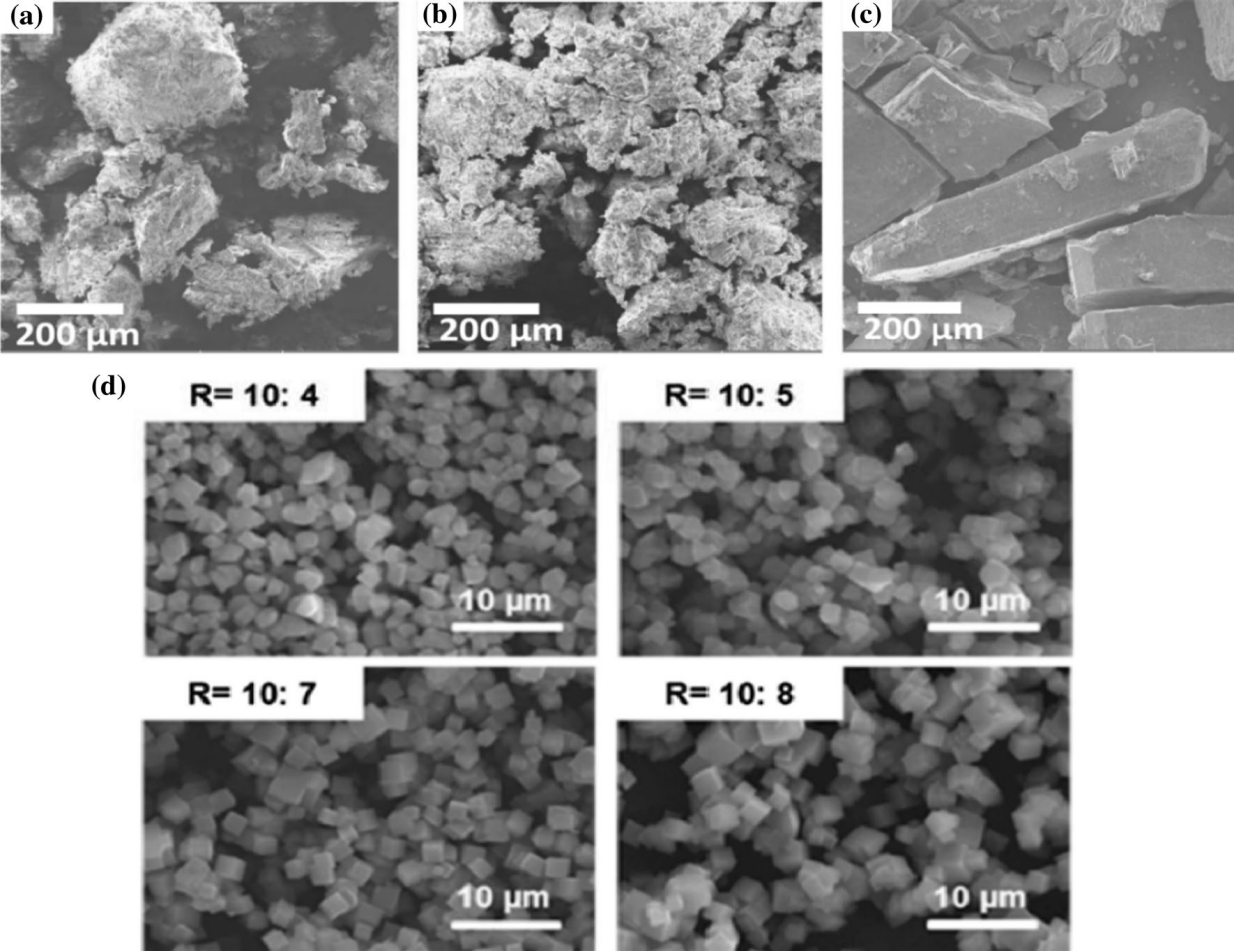
Scanning electron microscopy images of the as-fabricated potassium cations-β-cyclodextrin-based metal–organic framework by acetone, acetonitrile, and methanol (Volkova et al. 2020) and scanning electron microscopy of the as-fabricated γ-cyclodextrin-based metal–organic framework with different methanol ratios (Liu et al. 2017a). Copyright, 2022, ACS

In that connection regarding the anti-inflammatory drugs, Bernini et al. (2014) examined the capability of the potassium cations-β-cyclodextrin-based metal–organic framework to load ibuprofen. The incorporation of ibuprofen into the potassium cations-β-cyclodextrin-based metal–organic framework was executed via co-crystallization and impregnation protocols. The co-crystallization protocol's unsuitability was deduced since the ibuprofen loading was less than 1 wt%. While the ibuprofen loading by the impregnation protocol was 7.4 wt%, revealing advanced results compared to the co-crystallization protocol. Nonetheless, the ibuprofen loading within was quite higher, reaching 23–26 wt%, which may be attributed to the higher surface area of a γ-cyclodextrin-based metal–organic framework than β-cyclodextrin-based metal–organic framework since the uptake of ibuprofen is mainly controlled by the surface area of the metal–organic frameworks and the dimensions of the pores (Bernini et al. 2014). The influence of the anti-solvent on the crystallization of the potassium cations-β-cyclodextrin-based metal–organic framework was evaluated utilizing bountiful anti-solvent such as acetone, acetonitrile, and methanol. The results clarified that using acetonitrile instead of methanol prolonged the crystallization time from 96 to 192 h (Volkova et al. 2020). Conversely, the utilization of acetone shortened the crystallization time to 8 h, most likely due to the high vapor pressure of acetone, the diffusion rate into the reaction mixture of potassium cations-β-cyclodextrin-based metal–organic framework was enhanced. However, such a short crystallization time leads to a low crystallinity since the high volatile nature of acetone forms plenty of nuclei that rapid growth. Scanning electron microscopy images (Fig. 7A–C) of the as-fabricated potassium cations-β-cyclodextrin-based metal–organic framework by acetone, acetonitrile, and methanol proved that methanol is more appropriate than acetone and acetonitrile. Interestingly, Liu et al. (2017a) deduced that the ratio of methanol greatly impacted the morphology of the cyclodextrin-based metal–organic frameworks. Scanning electron microscopy image (Fig. 6D) revealed the transformation of γ-cyclodextrin-based metal–organic framework from irregular hexagonal to uniform cubic structure when the methanol ratio increased, inferring the contribution of methanol in the metal–organic framework nucleation (Liu et al. 2017a).

In another study, Hu et al. (2022b) evaluated the dominant parameters of the menthol capacity and the encapsulation efficiency of menthol within the β-cyclodextrin-based metal–organic framework. Hu et al. (2022b) recorded that increasing the encapsulation temperature from 65 to 90 ℃ enhanced the menthol capacity from 8 to 21.7% and the encapsulation efficiency from 6.3 to 23.6%, respectively. However, further increases in encapsulation temperature over 90 ℃ dwindled both encapsulation efficiency and menthol capacity (Hu et al. 2022b). The appropriate encapsulation temperature can explain these findings and acquires the menthol molecules more energy that facilitates interactions with the β-cyclodextrin-based metal–organic framework, thereby increasing the menthol capacity and encapsulation efficiency. While, the further raising in the encapsulation temperature accelerates the gas molecules' motion, decreasing the captured quantity of menthol in the β-cyclodextrin-based metal–organic framework (Fergoug et al. 2003). The volatile nature of menthol requires an adequate encapsulation time for melting and penetrating β-cyclodextrin-based metal–organic framework, so the impact of the encapsulation time of menthol into β-cyclodextrin-based metal–organic framework was thoroughly inspected (Tesfay et al. 2020). The result exhibited an amelioration in the menthol capacity and encapsulation efficiency with the increase in time reaching 25.8% and 32%, respectively, during 1 h, which may be due to the increase in the number of collisions between menthol and β-cyclodextrin-based metal–organic framework with the time. Contrariwise, when the encapsulation time increased than 1 h, a noticeable decline in the menthol capacity and encapsulation efficacy occurred since the possibility of menthol molecules escaping from the β-cyclodextrin-based metal–organic framework matrix increases with the increase in the time of encapsulation (Zhang et al. 2012).

Moreover, mixing and grinding encapsulation approaches were utilized to examine the impact of the encapsulation method on menthol capacity and encapsulation efficacy. The grinding efficiency surpassed the mixing approach since encapsulation efficacy and menthol capacity by grinding were 1.5 times greater than the mixing approach. Such behavior may be assigned to the higher ability of the grinding method to increase the contact between β-cyclodextrin-based metal–organic framework and menthol compared to the mixing method (Ghodki and Goswami 2016). Moreover, it was concluded that the β-cyclodextrin-based metal–organic framework possessed a high affinity for menthol since the maximum Menthol capacity was 30.60%. This result was in line with Hu et al. (2021) study that inferred the high affinity of a β-cyclodextrin-based metal–organic framework for menthol (21.76%) compared to the α-cyclodextrin-based metal–organic framework (5%) and γ-cyclodextrin-based metal–organic framework (11%), which may be related to the suitability of the pores size of β-cyclodextrin-based metal–organic framework to host menthol molecules (Hu et al. 2021). In yet another attempt, Liu et al. (2017c) fabricated Cs-β-cyclodextrin-based metal–organic frameworks via the template-induced method using 1,2,3-triazole-4,5-dicarboxylic acid, and methyl benzene sulfonic acid as template agents for β-cyclodextrin-based metal–organic framework-1 and β-cyclodextrin-based metal–organic framework-2, respectively (Liu et al. 2017c). Both β-cyclodextrin-based metal–organic framework-1 and β-cyclodextrin-based metal–organic framework-2 revealed superb performances as drug vehicles for 5-fluorouracil and methotrexate. The 5-fluorouracil loading content within β-cyclodextrin-based metal–organic framework-1 and β-cyclodextrin-based metal–organic framework-2 were 1.379 and 1.510 g/g, respectively, revealing an enhanced performance compared to mesoporous silica (Qu et al. 2006) and other metal–organic frameworks (Horcajada et al. 2012).

While the methotrexate loading content within β-cyclodextrin-based metal–organic framework-1 and β-cyclodextrin-based metal–organic framework-2 were 0.689 and 1.217 g/g, respectively, furthermore, the release rate values of 5-fluorouracil were 89 and 96.4%, and about 41.5 and 82.4% were released from β-cyclodextrin-based metal–organic framework-1 and β-cyclodextrin-based metal–organic framework-2, respectively. This result indicated the favorability of β-cyclodextrin-based metal–organic framework-1 over β-cyclodextrin-based metal–organic framework-2 owing to the smaller pores size of β-cyclodextrin-based metal–organic framework-1 (Liu et al. 2017c). In a similar study regarding the 5-fluorouracil within the cyclodextrin-based metal–organic framework, Sha et al. (2016) fabricated α-cyclodextrin-based metal–organic frameworks via vapor deposition and solvothermal approaches for drug delivery applications. Sha et al. (2016) found that the 5-fluorouracil loading within α-cyclodextrin-based metal–organic framework-1 by 0.257 g/g was higher than α-cyclodextrin-based metal–organic framework-2 by 0.107 g/g owing to the larger cavities of α-cyclodextrin-based metal–organic framework-1. Notably, 5-fluorouracil showed a fast release with a quite low cumulative release within 6 h was 77% for α-cyclodextrin-based metal–organic framework-1 and 79% for α-cyclodextrin-based metal–organic framework-2. The partial retention of 5-fluorouracil molecules may explicate this performance in the α-cyclodextrin-based metal–organic frameworks cavities since they could not transit the cavities to reach the solution. The cytotoxicity of α-cyclodextrin-based metal–organic frameworks was scrutinized on HepG2 cells, revealing the nontoxicity of both HepG2 cells (Sha et al. 2016).

To summarize, α-cyclodextrin, β-cyclodextrin and γ-cyclodextrin-based metal–organic frameworks can be considered outstanding green drug vehicles owing to their remarkable drug loading, sustainable drug release behavior, biocompatibility, easy fabrication, and eco-friendly advantage (Table 3). However, during our literature investigation, we noticed a scarcity of research papers involving the utilization of α-cyclodextrin-based metal–organic frameworks as drug carriers. Hence, we do recommend the implementation of additional studies regarding the evaluation of the drug delivery performance of α-cyclodextrin-based metal–organic frameworks.

**Table 3:** Utilization of cyclodextrin-based metal–organic frameworks and cyclodextrin-composites as drug vehicles

CD-MOF/ CD-MOF composites	CD	Metal salt	Metal ion	Synthesis method	Drug	Loading capacity	References
Nano ssCGP	γ-CD	KOH	K^+^	Vapor diffusion	Doxorubicin	45 mg/g	Xue et al. (2019)
RGD-functionalized CD-MOF	γ-CD	KOH	K^+^	Vapor diffusion	Doxorubicin	14%	Chen et al. (2021a)
Micro/nano cubic DPC-γ-CD-MOF sponges	γ-CD	KOH	K^+^	Vapor diffusion	Doxorubicin	60 – 80 mg/g	Singh et al. (2017)
γ-CD-MOF	γ-CD	KOH	K^+^	Vapor diffusion	Folic acid	35 wt%	Xu et al. (2019)
K-γ-CD Na-γ-CD Fe-γ-CD	γ-CD	KOH NaCl FeCl_3_	K^+^ Cl^−^ Fe^3+^	Vapor diffusion	Diclofenac sodium	50% 49% 55%	Abuçafy et al. (2018)
γ-CD-MOF	γ-CD	KOH	K^+^	Vapor diffusion	Florfenicol Enrofloxacin	54.60 mg/g 45.25 mg/g	Wei et al. (2021)
CD-MOF	γ-CD	KOH	K^+^	Vapor diffusion	Ibuprofen	23 – 26 wt%	Hartlieb et al. (2017)
γ-CD-MOF-b	γ-CD	KOH	K^+^	Vapor diffusion	Acetaldehyde	30 µg/g	Al-Ghamdi et al. (2016)
γ-CD-MOF	γ-CD	KOH	K^+^	Vapor diffusion	Methotrexate	~ 6 wt%	Kritskiy et al. (2020)
CD-MOF-Micro CD-MOF-Nano	γ-CD	KOH	K^+^	Microwave-assisted	Sucralose	17.5% 27.9%	Lv et al. (2017)
CD-MOF-1	γ-CD	KOH	K^+^	Microwave-assisted	Fenbufen	196 mg/g	Liu et al. (2017a)
Nano-γ-CD-MOF	γ-CD	KOH	K^+^	Vapor diffusion	18β-glycyrrhetinic acid	17.2%	Liu et al. (2022)
KAc-γ-CD-MOF (dense) KAc-γ-CD-MOF (porous)	γ-CD	KAc	K^+^	Two steps 1. Hydrothermal synthesis 2. Crystal transformation	Valsartan	33.5% 30.2%	Ding et al. (2019)
CD-MOF	γ-CD	KOH	K^+^	Vapor diffusion	Scutellarin	30.42%	Zhao et al. (2020b)
γ-CD-MOF	γ-CD	KNO_3_	K^+^	Vapor diffusion	Menthol	~ 11%	Hu et al. (2021)
KAc-γ-CD-MOF-THY-HT KOH-γ-CD-MOF-THY-HT KCl-γ-CD-MOF-THY-HT	γ-CD	KAc KOH KCl	K^+^	Hydrothermal	Thymol	293.8 mg/g 287.7 mg/g 249.3 mg/g	Pan et al. (2022)
β-CD-MOF	β-CD	Na_2_C_2_O_4_	Na^+^	Solvothermal	5-Fluorouracil	23.02%	Lu et al. (2015)
β-CD-MOF-1 β-CD-MOF-2	β-CD	CsCl	Cs^+^	Template-induced approach	5-Fluorouracil Methotrexate 5-Fluorouracil Methotrexate	1.379 g/g 0.689 g/g 1.510 g/g 1.217 g/g	Liu et al. (2017c)
β-CD-MOF	β-CD	KOH	K^+^	Hydrothermal	Naringin	82.3%	Li et al. (2021a)
β-CD-MOF-IC	β-CD	KOH	K^+^	Vapor diffusion	Menthol	30.60%	Hu et al. (2022b)
β-CD-MOF	β-CD	KOH	K^+^	Vapor diffusion method	Quercetin Emodin	103.6 mg/g 177.26 mg/g	Kong et al. (2019)
β-CD-MOF	β-CD	KOH	K^+^	Vapor diffusion	Ibuprofen	7.4 wt%	Volkova et al. (2020)
SHPs@β-CDMOF β-CD-MOF@SHPs	β-CD	KCl	K^+^	Vapor diffusion	Curcumin	1.78% 1.49%	Shao et al. (2022)
K- β-CD -MOF Cs- β-CD -MOF	β-CD	KOH	K +	Vapor diffusion	Myricetin	282.39 mg/g 308.65 mg/g	Jiang et al. (2021)
β-CD -MOF	β-CD	KNO_3_	K^+^	Vapor diffusion	Menthol	21.76%	Hu et al. (2021)
β-CD-MOF	β-CD	KOH	K^+^	Vapor diffusion Solvothermal	Dimercaptosuccinic acid	15%	Xiong et al. (2019)
α-CD-MOF-1 α-CD-MOF-2	α-CD	KOH	K^+^	Vapor diffusion Solvothermal	5-Fluorouracil	0.257 g/g 0.107 g/g	Sha et al. (2016)
α-CD-MOF	α-CD	KNO_3_	K^+^	Vapor diffusion	Menthol	~ 5%	Hu et al. (2021)

The synthesis methods and materials of CD-MOF/ CD-MOF composites are summarized. Potential loaded drugs and the loading capacity of each CD-MOF/ CD-MOF composite are explained. CD-MOF refers to a cyclodextrin-based metal–organic framework, CD refer to cyclodextrin, ssCGP refers to novel cubic gel particles, KAc refers to Potassium acetate, KOH refers to potassium hydroxide, DPC refers to diphenyl carbonate-crosslinked, and SHPs refer to soybean hull polysaccharides

### Cyclodextrin-based metal–organic frameworks as sensors

**Fig. 8:**
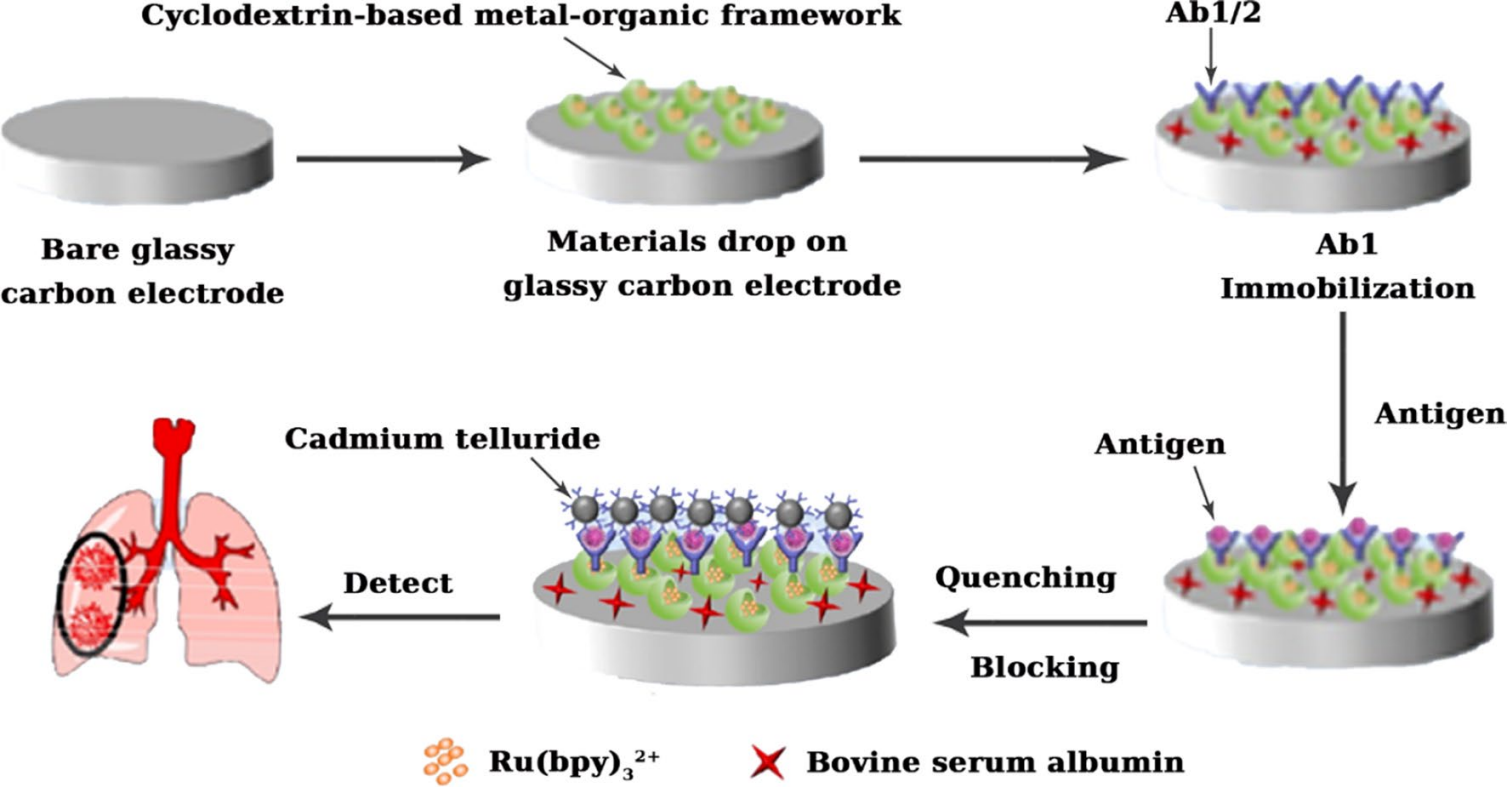
Fabrication of cyclodextrin-based metal–organic framework@Ru(bpy)_3_^2+^ nano-sensor for the cytokeratin 19 fragments antigen 21–1 detection. The process involves cyclodextrin-based metal–organic frameworks

Cyclodextrin-based metal–organic frameworks have attracted much research interest as promising sensor-based materials because of their outstanding absorption properties, biocompatibility, and interactions with targeted materials (Li et al. 2021b). Recently, Ru(bpy)_3_^2+^-encapsulated cyclodextrin-based metal–organic framework have been proposed and developed by Wang et al. (2021a) as a sandwich-type electrochemiluminescent biosensor where cyclodextrin-based metal–organic framework with excellent biocompatibility and high surface area were blended with Ru(bpy)_3_^2+^ that possesses outstanding electrochemiluminescence features (Fig. 8). Ru(bpy)32 + -encapsulated cyclodextrin-based metal–organic framework as biosensor showed high stability, low detection limit, and excellent selectivity for the electrochemiluminescence determination of cytokeratin-19 fragment antigen 21–1 in A549 lung cancer cells. Moreover, a novel β-cyclodextrin-stabilized metal–organic framework-235 hybrid has been reported as an effective catalyst in hydrogen peroxide-luminol chemiluminescence reaction for glucose detection (Mao et al. 2018). Metal–organic framework-235/β-cyclodextrin recorded greater than 30-fold improvement in chemiluminescence of the hydrogen peroxide-luminol system compared with that of hydrogen peroxide-luminol. Metal–organic framework-235/β-cyclodextrin showed excellent sensitivity, a broad linear range of 0.01–3, a low detection limit of 10 nM for glucose, perfect constancy, and accuracy of analysis of real biologically obtained samples attributed to combined characteristics of β-cyclodextrin and metal–organic framework-235 (Mao et al. 2018).

Interestingly, the presence of various functional groups in cyclodextrin-based metal–organic framework composites make them good candidates for recognizing detectable biomarkers where cyclodextrin-based metal–organic framework composites easily anchor nucleic acids. In that respect, Jalili et al. (2019) prepared a dual-emissive metal–organic framework composite by simultaneously introducing blue and yellow light-emitting into zeolitic imidazolate framework-8 for glutathione detection. After the sensing pathway applying metal–organic frameworks and cyclodextrins as co-receptor, Cu^2+^ ions were included in the developed biosensor to connect the amino group of yellow cyclodextrins and the carboxyl group of blue cyclodextrins (Jalili et al. 2019). Jalili et al. (2019) also found that the transfer of electrons between Cu^2+^ and amino groups of yellow cyclodextrins could promote the yellow fluorescence, while there was a diminishing of blue fluorescence upon the coordination between Cu^2+^ and the carboxyl group groups of blue cyclodextrins. When glutathione was added to the Cu^2+^/BY cyclodextrins@zeolitic imidazolate framework-8 system, it interacted strongly with Cu^2+^ causing a decrease in Cu^2+^ amount and breaking the link between the cyclodextrins and Cu^2+^, leading to restoring the fluorescence of both carbon dots gradually to their initial state. Glutathione was calculated in the range of 3–25 nM with a detection limit of 0.90 nM under the optimal conditions (Jalili et al. 2019).

In another investigation, Mxene/Carbon nanohorns/β-cyclodextrin-based metal–organic frameworks were fabricated as rapid and sensitive electrochemical sensors for carbendazim detection (Tu et al. 2020). The fabricated composites possessed great adsorption efficiency for detecting targeted materials owing to β-cyclodextrin-based metal–organic frameworks' high porosity. The linear range of the fabricated platform was 3.0 nM–10.0 µM a with a detection limit of 1.0 nM. MXene/Carbon nanohorns/β-cyclodextrin-based metal–organic frameworks electrode exhibited a wide linear range and low limit of detection due to the combination of the two materials; the MXene/Carbon nanohorns possessed great electronic conductivity and high surface area, and an abundance of active positions. β-cyclodextrin-based metal–organic frameworks have high porosity and pore volume of metal–organic frameworks, resulting in high adsorption efficiency for carbendazim (Tu et al. 2020). Baicalin is a significant biologically active phenolic flavonoid compound that supplies a great opportunity for more advancements to discover new drugs for treating Coronavirus disease 2019 (Zandi et al. 2021).

However, the quantity of baicalin must be controlled accurately; otherwise, a higher dose of baicalin could cause health problems like diarrhea, muscle soreness, leukopenia, and low-grade fever. With the merits of accuracy and sensitivity, a novel and highly sensitive baicalin sensor with high performance based on carbon-nitride-single-walled carbon nanotube nanocomposites/reduced graphene oxide/cyclodextrin-based metal–organic framework nanocomposite was developed by Zhao et al. (2022b). Zhao et al. (2022b) found that the sensor obtained a superior linear range of 1 × 10^–9^ to 5 × 10^–7^ M with a limit of detection of 4.6 × 10^–10^ M and a sensitivity of 220 A.M^−1^. To enhance the detection performance for baicalin, Zhao et al. (2022b) have adopted a synergistic pathway for detecting through the high catalytic properties of carbon-nitride and the large electrochemical properties of single-walled carbon nanotube nanocomposites with a large surface area and stability and biocompatibility of reduced graphene oxide. In addition, baicalin can be adsorbed by a cyclodextrin-based metal–organic framework on the electrode surface owing to the outstanding enrichment capability of the cyclodextrin-based metal–organic framework, making carbon-nitride-single-walled carbon nanotube nanocomposites/reduced graphene oxide/cyclodextrin-based metal–organic framework promising sensory material for the detection of flavonoids (Zhao et al. 2022b).

To summarize, cyclodextrin-based metal–organic frameworks are promising sensor-based materials due to their exceptional absorption properties, biocompatibility, and interactions with targeted substances. β-cyclodextrin-based metal–organic framework-235 recorded a higher than 30-fold improvement in chemiluminescence of the hydrogen peroxide-luminol system compared with that of hydrogen peroxide-luminol. Ru(bpy)32 + -encapsulated cyclodextrin-based metal–organic framework as biosensor showed high sensitivity, excellent stability, and low detection limit for the electrochemiluminescence determination of cytokeratin-19 fragment antigen 21-1 in A549 lung cancer cells.

### Cyclodextrin-based metal–organic frameworks as adsorbents

Adsorption is a highly efficient technique and the most used method in many fields, such as wastewater treatment and air pollution. Adsorption capacity depends on many factors such as adsorbent nature, surface area, temperature, pressure, and contact time. Cyclodextrin-based metal–organic frameworks have been widely applied in separation and adsorption applications due to cyclodextrin-based metal–organic frameworks' porosity, crystalline structure, stability, and complexation ability (Roy and Stoddart 2021). Daily releasing carbon dioxide from human and industrial activities, fuel combustion, and natural sources produce global warming. Carbon sequestration is one of the most achievable techniques to reduce the emissions of carbon dioxide released from anthropogenic and industrial activities. Due to the pore size of 1.9 nm, large surface area, and high stability of modified cyclodextrin-based metal–organic frameworks, modified cyclodextrin-based metal–organic frameworks are ideal candidates for carbon capture. Starting with renewable γ-cyclodextrin and through vapor diffusion at room temperature, a cyclodextrin-based metal–organic framework was synthesized, which shows great adsorption capacity for carbon dioxide to reach 24 mg-carbon dioxide/g-cyclodextrin-based metal–organic frameworks through hydroxyl groups within the γ-cyclodextrin, carbon dioxide captured, and formed carbonic acid investigated by the color change for pH-responsive. In another investigation, cubic crystals of potassium bicarbonate cyclodextrin-based metal–organic framework synthesized underlying solvothermal conditions at 120–160 °C, which represented excellent selectivity, stability, and high adsorption capacity for carbon dioxide reached 2.78 mmol g^−1^, 1 bar carbon dioxide at 30 °C in volumetric measurements (Zick et al. 2022).

On the other hand, amino groups were introduced to the β-cyclodextrin-based metal–organic framework to enhance the carbon dioxide capture capacity of the β-cyclodextrin-based metal–organic framework from 1.2 to 12.3 cm^3^/g at 273 K as well as enhancing the water stability from 18 to 24 mg/mL, and this is due to the polar nature of β-cyclodextrin (Xu et al. 2020). Moreover, a γ-cyclodextrin-based metal–organic framework with crystalline morphology and surface area reaching 775 m^2^/g and a pore volume of 0.229 cm^3^/g was synthesized by vapor diffusion method at high temperature and pressure (Hamedi et al. 2021). Hamedi et al. (2021) found that the carbon dioxide capture increased with raising pressure and lowering. Besides, the maximum carbon dioxide capture of γ-cyclodextrin-based metal–organic framework reached as high as 326 mg/g at 303 K. Moreover, Wang et al. (2018) prepared γ-cyclodextrin-based metal–organic framework-potassium with excellent adsorption capacity for formaldehyde. The high adsorption capacity of γ-cyclodextrin-based metal–organic framework-potassium was attributed to the porous structure, high surface area, and hydrogen bonds formed between the hydroxyl group provided by the metal–organic framework surface and formaldehyde, which suggested the high adsorption capacity for polar compounds. Importantly, Wang et al. (2018) found that the metal ion does not influence the adsorption capacity where both γ-cyclodextrin-based metal–organic framework-caesium and γ-cyclodextrin-based metal–organic framework-potassium showed almost similar adsorption capacity for formaldehyde with little change in the crystalline shape.

To summarize, modified cyclodextrin-based metal–organic frameworks are ideal candidates for carbon capture because of the pore size of 1.9 nm, large surface area, and high stability of modified cyclodextrin-based metal–organic frameworks. Cyclodextrin-based metal–organic framework showed great carbon dioxide adsorption capacity, reaching 24 mg-carbon dioxide/g-cyclodextrin-based metal–organic frameworks through hydroxyl groups. Besides, γ-cyclodextrin-based metal–organic framework-potassium with excellent adsorption capacity for formaldehyde.

### Cyclodextrin-based metal–organic frameworks for gas separation

Mehmood et al. (2020) prepared defect-free cellulose acetate-based mixed matrix membranes modified with γ-cyclodextrin and metal–organic framework using a solution casting technique for gas separation. Mehmood et al. (2020) investigated the permeation behavior of cellulose acetate/γ-cyclodextrin-based metal–organic framework membranes. The fabricated membranes were found to have dense and isotropic morphology with uniform filler dispersion with no cracks or agglomerations. The carbon dioxide/methane separation efficiency of cellulose acetate/γ-cyclodextrin-based metal–organic framework membranes were subjected to permeation tests using both single and mixed gas conditions. The permeability and selectivity of cellulose acetate/γ-cyclodextrin-based metal–organic framework membranes recorded a decreasing trend, that is, 36.79–35 of both carbon dioxide and methane was observed by increasing the filler weight percentage 0.2–1.0, and pressure ranged from 1 to 5 bar with the highest carbon dioxide/methane selectivity reached 38.49 (Mehmood et al. 2020).

In another study, Li et al. (2019) fabricated two isostructural cyclodextrin-based metal–organic frameworks, e.g., cyclodextrin-based metal–organic framework-1 and cyclodextrin-based metal–organic framework-2 for carbon dioxide/acetylene separation to remove carbon dioxide impurity and obtain the high purity acetylene that is desired by many industries. The two composites, cyclodextrin-based metal–organic framework-1 and cyclodextrin-based metal–organic framework-2, revealed superior adsorption capacity and selectivity (~ 119) for carbon dioxide/acetylene mixture at ambient temperature. This performance led to obtaining pure acetylene in one step (Li et al. 2019). Jin et al. (2018) have fabricated acetylene $\subset$ APPT-cyclodextrin-based metal–organic frameworks via acetylene uptake. The uptake mechanism of acetylene into APPT-cyclodextrin-based metal–organic frameworks with uncoordinated perchlorate and tetrafluoroborate was investigated by single-crystal X-ray diffraction. The strong affinity of cyclodextrin-based metal–organic frameworks for acetylene was found to be originated from the C–H···X (X = O and F) H-bonding interactions within the cyclodextrin-based metal–organic framework confined space (Jin et al. 2018).

To summarize, cyclodextrin-based metal–organic frameworks showed excellent properties for gas separation. Cyclodextrin-based metal–organic framework-1 and cyclodextrin-based metal–organic framework-2 revealed superior adsorption capacity, and selectivity for carbon dioxide/acetylene mixture at room temperature.

### Cyclodextrin-based metal–organic frameworks membranes

Cyclodextrins have been applied in many fields as a separation and adsorption membrane due to their unique properties and were introduced to the polyurethane matrix to isolate carbon dioxide from methane. Polyurethane/γ-cyclodextrin-based metal–organic frameworks show promising penetration and superlative selectivity for carbon dioxide because of the amino group and hydroxyl group provided by polyurethane and the physical nature of the reaction between polyurethane and γ-cyclodextrin-based metal–organic frameworks (Mehmood et al. 2020). Titanium dioxide nanoparticles were introduced into the polyethersulfone/β-cyclodextrin loose nanofiltration membrane through 1, 6-hexamethylene diisocyanate as a bridge to improve hydrophilic and separation properties of the membrane and applied for salts and dye removal from water. The fabricated membrane possessed a permeate flux for Congo red dye at about 30.5 L m^−2^ h^−1^ at 0.2 MPa and permeability for sodium sulfate at about 94.0%, revealing the promising candidate for wastewater treatment applications (Zhang et al. 2019b).

In another attempt, a novel phytic acid-β-cyclodextrin composite was synthesized through poly-condensation reaction introducing super adsorption capacities for cationic dyes, e.g., methylene blue was 1095 mg/g, astrazon pink FG was 2005.58 mg/g, and crystal violet was 1736.32 mg/g (Li et al. 2022). The multilayer of β-cyclodextrin decorated reduced graphene oxide complex membrane with a thickness of 310 nm represents permeation rates in 96 h for Evans blue was 0.31 × 10^–4^ mmol m^−2^ h^−1^, Methyl orange was 1.58 × 10^–4^ mmol m^−2^ h^−1^, and Rhodamine B was 0.70 × 10^–4^ mmol m^−2^ h^−1^, slightly could pass through the β-cyclodextrin decorated reduced graphene oxide complex membrane which attributed to dyes molecule size, on the other hand, size of hydrophobic nanochannels located between layers of β-cyclodextrin decorated reduced graphene oxide complex membrane play a vital rule in the blocking process of metal ions as with increase metal ions diameter they couldn't pass through nanochannels and blocked on the feed side (Cheng et al. 2022).

To summarize, due to the amino and hydroxyl groups provided by polyurethane and the physical nature of the reaction between polyurethane and γ-cyclodextrin-based metal–organic frameworks, polyurethane/γ-cyclodextrin-based metal–organic frameworks show promising penetration and superlative selectivity for carbon dioxide. Polyethersulfone/β-cyclodextrin loose nanofiltration membrane through 1, 6-hexamethylene diisocyanate as a bridge to improve hydrophilic and separation properties of the membrane and was applied to remove salts and dyes from water.

## Conclusion

Cyclodextrin-based metal–organic frameworks are composed of biocompatible metal ions including calcium, potassium, and titanium with cyclodextrin, including α-cyclodextrin, β-cyclodextrin, and γ-cyclodextrin through well-organized metal–ligand coordination bonds. Cyclodextrin-based metal–organic frameworks are a viable alternative to metal–organic frameworks due to their advantageous properties, such as non-toxicity, edibility, renewable nature, biodegradability, high specific surface area, and biocompatibility. Among cyclodextrin types, γ-cyclodextrin is a suitable type for preparing metal–organic frameworks with biocompatible and non-toxic properties. Consequently, cyclodextrin-based metal–organic frameworks have unlocked a new research path in several fields, particularly the food industry and drug delivery. In which cyclodextrin-based metal–organic frameworks can be used as versatile hosts to encapsulate sufficient biological agents and transform crystal drugs into a molecular state, thereby enhancing the stability, solubility, and bioavailability of poorly soluble drugs. Moreover, cyclodextrin-based metal–organic frameworks are considered promising carriers for antibacterial compounds in food packaging. Several synthesis techniques can be used to prepare cyclodextrin-based metal–organic frameworks, including vapor diffusion, microwave-assisted, hydro/solvothermal, and ultrasound. The vapor diffusion method is the most widespread method among previous synthesis techniques to synthesize cyclodextrin-based metal–organic frameworks, followed by hydro/solvothermal methods. Microwave-assisted hydro/solvothermal synthesis was the best technique for obtaining a rapid reaction and controlling particle size. In addition, regardless of the method used to synthesize cyclodextrin-based metal–organic frameworks, the choice of additives such as cetyltrimethylammonium bromide, menthol, polyethylene glycol, and so on, as assistant agents are crucial for both size control and their applications. As a non-toxic material, polyethylene glycol is the best additive that can be used to replace toxic cetyltrimethylammonium bromide. Before using cyclodextrin-based metal–organic frameworks in the food industry and drug delivery, the in vivo and clinical toxicity of different cyclodextrin-based metal–organic frameworks are required to evaluate their safety. In addition, other metal–organic framework synthesis techniques, such as ultrasonic, mechano-chemical, and electrochemical synthesis, may be worth attempting for the cyclodextrin-based metal–organic framework to further reduce particle size.
